# Systematic assessment of the replicability and generalizability of preclinical findings: Impact of protocol harmonization across laboratory sites

**DOI:** 10.1371/journal.pbio.3001886

**Published:** 2022-11-23

**Authors:** María Arroyo-Araujo, Bernhard Voelkl, Clément Laloux, Janja Novak, Bastijn Koopmans, Ann-Marie Waldron, Isabel Seiffert, Helen Stirling, Katharina Aulehner, Sanna K. Janhunen, Sylvie Ramboz, Heidrun Potschka, Johanna Holappa, Tania Fine, Maarten Loos, Bruno Boulanger, Hanno Würbel, Martien J. Kas

**Affiliations:** 1 Groningen Institute for Evolutionary Life Sciences, University of Groningen, Groningen, the Netherlands; 2 Animal Welfare Division, Vetsuisse Faculty, University of Bern, Bern, Switzerland; 3 Pharmalex, Mont-Saint-Guibert, Belgium; 4 Sylics (Synaptologics BV), Amsterdam, the Netherlands; 5 Institute of Pharmacology, Toxicology, and Pharmacy, Ludwig-Maximilians-Universitaet Muenchen, Muenchen, Germany; 6 Orion Pharma, Turku, Finland; 7 PsychoGenics Inc., New Jersey, Paramus, United States of America; 8 Teva Pharmaceuticals, Tel Aviv, Israel; Universidade Federal de Santa Catarina, BRAZIL

## Abstract

The influence of protocol standardization between laboratories on their replicability of preclinical results has not been addressed in a systematic way. While standardization is considered good research practice as a means to control for undesired external noise (i.e., highly variable results), some reports suggest that standardized protocols may lead to idiosyncratic results, thus undermining replicability. Through the EQIPD consortium, a multi-lab collaboration between academic and industry partners, we aimed to elucidate parameters that impact the replicability of preclinical animal studies. To this end, 3 experimental protocols were implemented across 7 laboratories. The replicability of results was determined using the distance travelled in an open field after administration of pharmacological compounds known to modulate locomotor activity (MK-801, diazepam, and clozapine) in C57BL/6 mice as a worked example. The goal was to determine whether harmonization of study protocols across laboratories improves the replicability of the results and whether replicability can be further improved by systematic variation (heterogenization) of 2 environmental factors (time of testing and light intensity during testing) within laboratories. Protocols were tested in 3 consecutive stages and differed in the extent of harmonization across laboratories and standardization within laboratories: stage 1, minimally aligned across sites (local protocol); stage 2, fully aligned across sites (harmonized protocol) with and without systematic variation (standardized and heterogenized cohort); and stage 3, fully aligned across sites (standardized protocol) with a different compound. All protocols resulted in consistent treatment effects across laboratories, which were also replicated within laboratories across the different stages. Harmonization of protocols across laboratories reduced between-lab variability substantially compared to each lab using their local protocol. In contrast, the environmental factors chosen to introduce systematic variation within laboratories did not affect the behavioral outcome. Therefore, heterogenization did not reduce between-lab variability further compared to the harmonization of the standardized protocol. Altogether, these findings demonstrate that subtle variations between lab-specific study protocols may introduce variation across independent replicate studies even after protocol harmonization and that systematic heterogenization of environmental factors may not be sufficient to account for such between-lab variation. Differences in replicability of results within and between laboratories highlight the ubiquity of study-specific variation due to between-lab variability, the importance of transparent and fine-grained reporting of methodologies and research protocols, and the importance of independent study replication.

## Abstract 2

The influence of protocol standardization between laboratories on their replicability of preclinical results has not been addressed in a systematic way. This study reveals differences in replicability of results within and between laboratories, highlighting the ubiquity of study-specific variation due to between-lab variability, the importance of transparent and fine-grained reporting of methodologies and research protocols, and the importance of independent study replication.

## Introduction

In recent years, the scientific community has raised concerns about the replicability of results, particularly in the preclinical biomedical sciences. Defining results replicability as the ability to duplicate results from a previous scientific claim supported by new data [1,2]. Various causes of poor replicability have been proposed, including the diverse methodologies used in the field and the lack of rigorous research practices (e.g., underpowered studies, risks of biases, inadequate statistics) [3–7]. Although these causes can certainly explain part of the problem, they permeate different science subfields differently [8] and cannot account for the poor replicability of results on their own. To our knowledge, no systematic studies have been performed to investigate the effect of protocol standardization within laboratories and protocol harmonization across laboratories regarding between-laboratory variation in light of replicability and generalizability of results.

The current and most common research practice of conducting single laboratory studies under standardized conditions has recently been proposed as a source of the high variability of results between laboratories [9,10]. Whenever rigorous standardization of environmental conditions within a study leads to homogenous study populations, the study results may become idiosyncratic as the study population is only representative of the narrow set of conditions in which it was tested. This increases the risk of replication failure even under only slightly different conditions as standardized; such single-site study designs do not allow predicting changes in the expression of the phenotype in response to different environmental influences. The change in the expression of the phenotype is caused by biological variation [11], which describes how genetic variation interacts with environmental factors to which experimental animals are exposed throughout development (gene–environment interactions), thereby shaping their phenotype [12].

Another approach taken to deal with the variability of results across laboratories is to harmonize the same standardized protocol across studies [13]. If harmonization includes those environmental and experimental factors that may influence the phenotype expression, it should result in replicable results. However, current evidence is ambiguous. Whereas in one study a rigorously standardized protocol that was harmonized across 3 laboratories resulted in many nonreplicable findings [14], another study that also followed protocol standardization and harmonization across 3 sites found similar phenotypic and pharmacological effects; however, the proportion of variation explained by lab was not formally assessed [15]. This suggests that this experimental approach may be missing to address some unknown source of variability between sites.

Certainly, there are inherent differences between laboratory environments that are not addressed in multi-laboratory protocols because of the low feasibility of harmonizing them or simply because these differences are not known (e.g., different ways to handle the animals, diversity in equipment). Some of these differences likely interact with the phenotype expression; this interaction may be accentuated when other sources of variability are minimized (i.e., standardized). Thus, although the same standardized protocol is implemented in different sites, it may still produce different results [16]. Still, there are no accounts to evaluate the impact that protocol harmonization across sites has on between-lab variability.

Furthermore, it has been recently suggested that if the between-lab variation can be incorporated within a single lab, the replicability of results between studies would increase [17–19]. Such an approach has been previously implemented [17,19–21]; yet, it has not been compared to a nonharmonized study across laboratories to assess the effect on between-lab variation.

To shed light on the effects of protocol harmonization across laboratories, we studied on one side whether harmonization of a standardized protocol reduces between-lab variation in comparison to a nonharmonized local protocol. Furthermore, we tested the effect of systematic heterogenization to assess whether within-lab heterogenization can further reduce between-lab variation compared to the standardized protocol. The experiments performed in this paper are defined as knowledge-claiming research according to Bespalov and colleagues [22].

## Results

### Stage 1: Local protocol

In this stage, 2 different compounds with opposite effects were tested to assess their effect on the distance traveled in the OF across the 7 sites. The 3 mg/kg Diazepam group showed strong sedative effects (i.e., no distance traveled) relative to its control group; this made the comparison across treatments and laboratories uninformative given the floor effect (Tables A and B in S1 Supplementary Stage). Therefore, the analysis of results was focused on the effects of MK-801.

**Fig 1:**
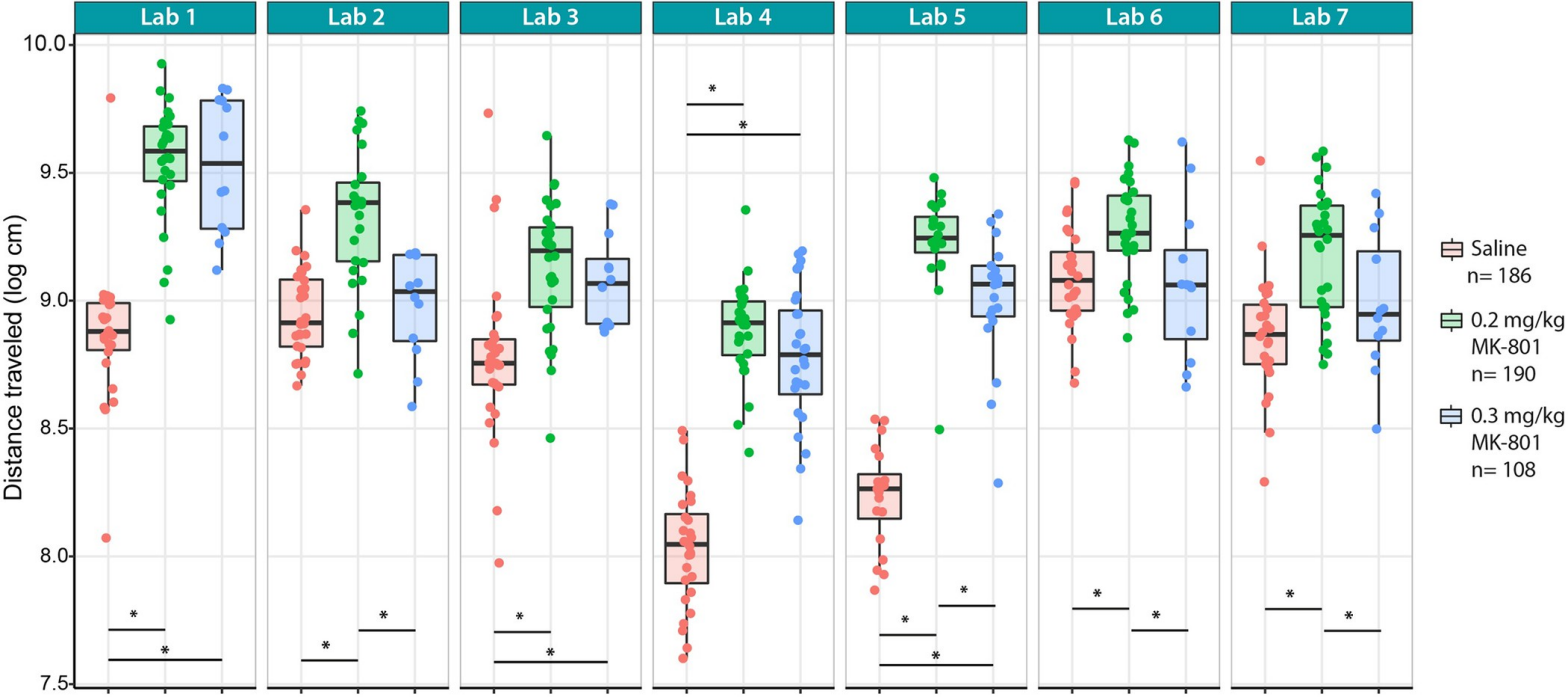
Tukey box plots and individual data points of the total distance traveled (log-transformed) in a 15-minute open field across 7 laboratories. All labs reported significant differences (*p < 0.05) between the groups receiving saline (red symbols) and 0.2 mg/kg MK-801 (green symbols). Labs 1, 3, 4, and 5 also found significant differences between animals receiving 0.3 mg/kg MK-801 (blue symbols) and saline. In addition, labs 2, 5, 6, and 7 found significant differences between the 2 different drug treatments of MK-801. Data underlying this figure can be found in https://osf.io/8f6yr/, Stage 1 folder.

**Fig 2:**
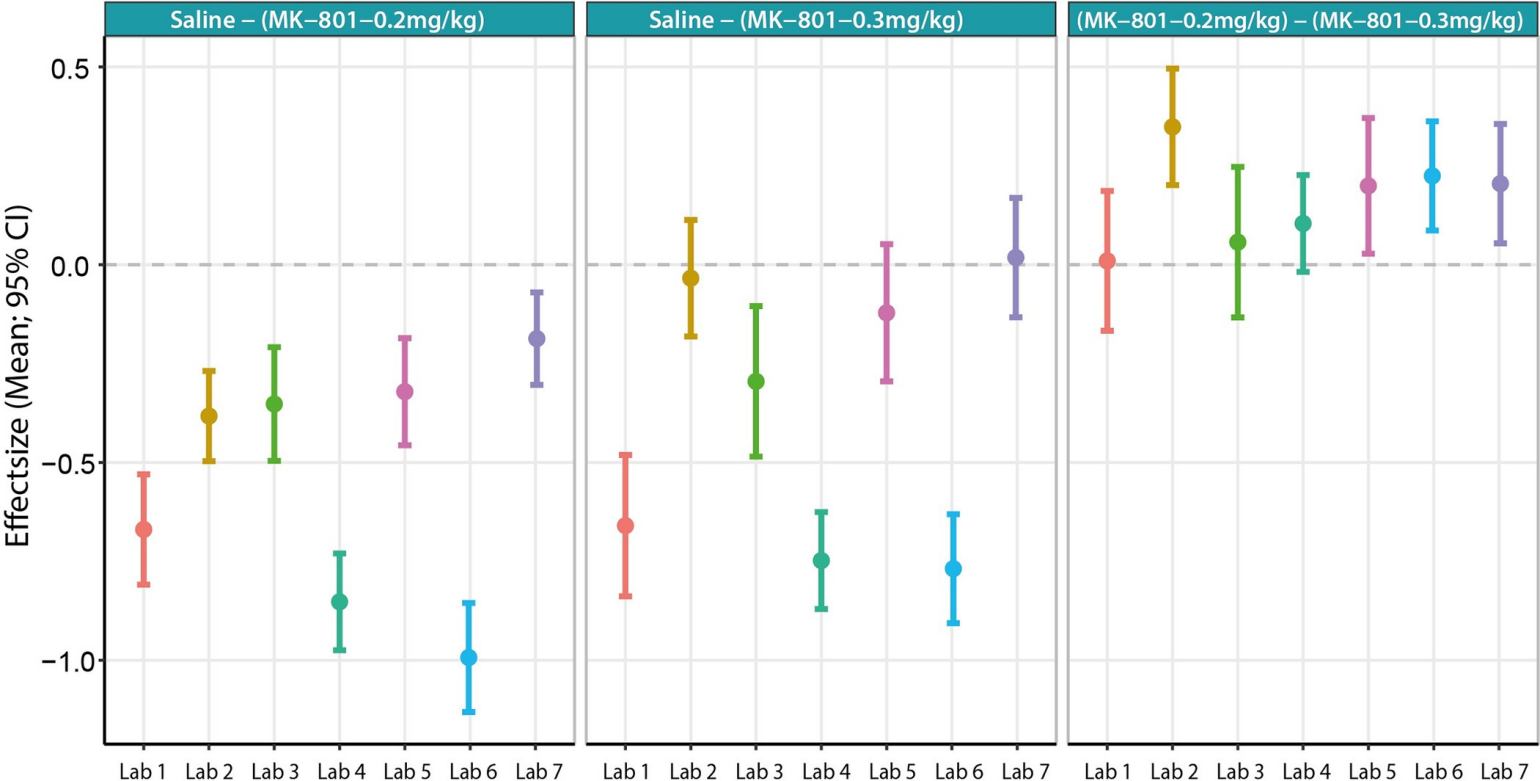
Mean and 95% CI of the treatment effect differences across laboratories between saline and MK-801 0.2 mg/kg (left panel), between MK-801 0.3 mg/kg (middle panel) and comparing both MK-801 drug treatments with each other (right panel). Data underlying this figure can be found in the Table A in S1 Supplementary Stage.

The local protocol showed a significant drug treatment effect with 0.2 mg/kg MK-801 increasing locomotion compared to saline treatment; this was replicated across all sites (Fig 1). When looking closely at this effect, although all sites found a significant effect, effect size differed across sites. On the other hand, the treatment with 0.3 mg/kg MK-801 drug treatment on distance moved for all 7 sites were into the same direction; however, based on statistical findings, only 4 out of 7 sites found a significant increase in distance moved (Fig 2). All the statistical results of the analysis of the treatment effect per laboratory can be found in Table A in S1 Supplementary Stage.

The comparison of results across laboratories (model 2) revealed that one-third (33%) of the total variance was associated with differences between laboratories. The interaction of the drug treatment effects and the laboratory explained 25% of the variance, while the remaining 41% of the variance was attributed to the residual (Table 1).

**Table 1:** Variance components of the across-laboratory analysis (model 2).

Parameter	Variance estimate	%
Lab	0.045	33.19
DrugTreatment:Lab	0.034	25.23
Residual	0.057	41.58

### Stage 2: Local and harmonized (standardized and heterogenized) protocols

**Fig 3:**
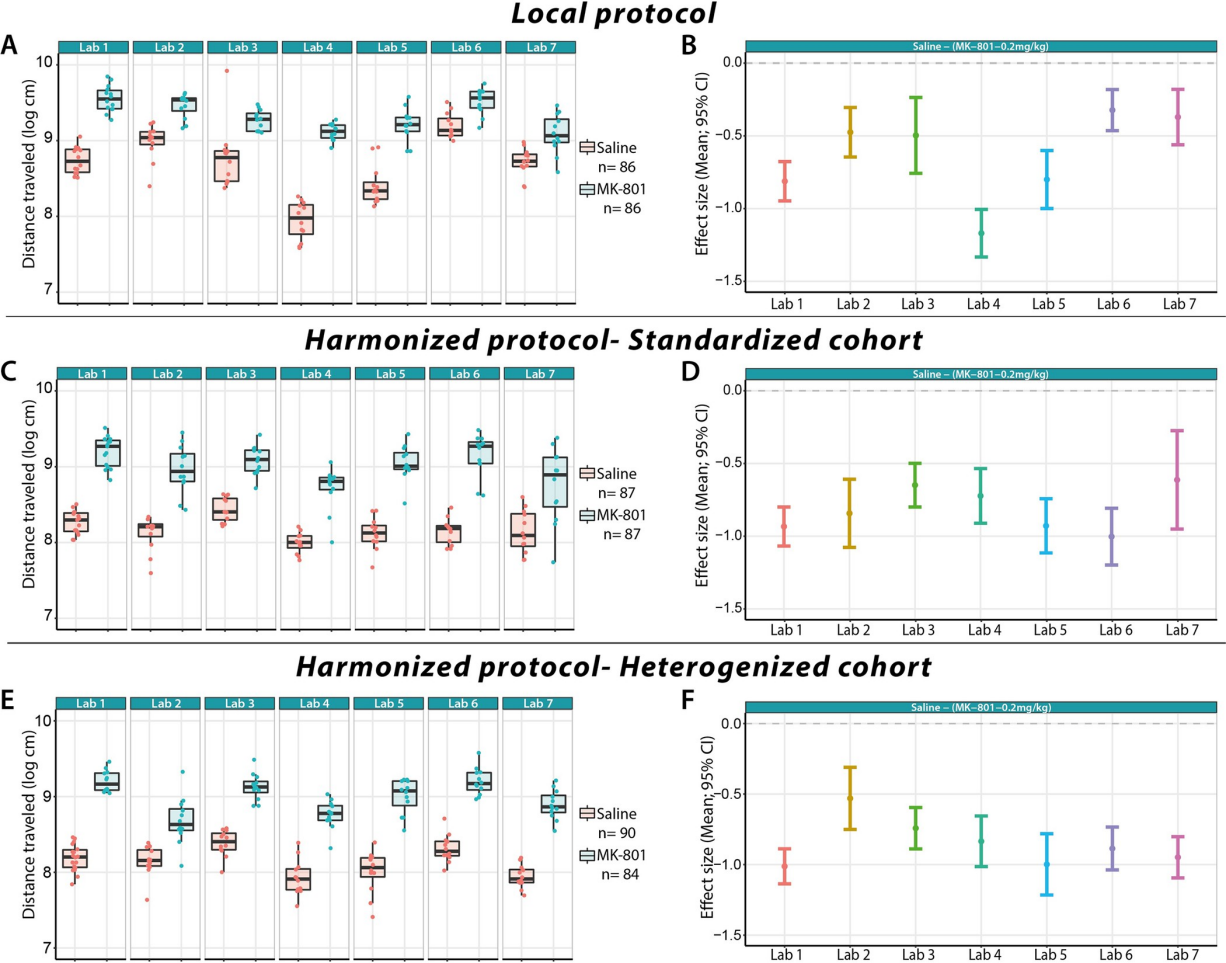
Box-plots and individual data points of the total distance traveled (left column) and treatment effect differences (right column) for the different protocols used in stage 2: (**A**, **B**) Local, (**C**, **D**) Harmonized-Standardized cohort, and (**E**, **F**) Harmonized-Heterogenized cohort. All sites found statistical differences (*p* < 0.05) in the distance traveled after saline (teal symbols) and 0.2 mg/kg MK-801 (red symbols) treatments. Data underlying this panels A, C, and E can be found in https://osf.io/8f6yr/, Stage 2 folder. Data underlying panels B, D, and F can be found in Tables A, B, and C, respectively, within S2 Supplementary Stage.

This stage aimed to assess the impact of harmonization of protocols across sites, and heterogenization of protocols within sites, on the replicability of the results. Therefore, standardized and heterogenized cohorts of the harmonized protocol were compared with the local protocol across the 7 sites in terms of between-laboratory variation in results (Fig 3). First, we evaluated the drug treatment effect in each laboratory for each of the protocols (model 1, Tables A, B, and C in S2 Supplementary Stage). When comparing the treatment effects in each of the laboratories, the analysis revealed that for the 3 protocols, the 0.2 mg/kg MK-801 resulted in a significantly larger distance traveled than the saline condition.

For each protocol, we found that the variance results from the Local protocol of stage 1 were highly similar to the variance results in stage 2, suggesting replicability when each laboratory followed its own protocol. Variance components for this protocol in both stages are similar and represent the same proportions (Table D in S2 Supplementary Stage). In addition, the across stages model (model 3) shows that the variability induced by the stage is nearly 0 (Table 2).

**Table 2:** Across-stage comparison of the Local protocol in stages 1 and 2 (model 3).

Parameter	Std. Dev.	Variance Estimate	%
DrugTreatment:Stage	0.041	0.002	1.12
Stage	0.000	0.000	0.00
DrugTreatment:Lab	0.202	0.041	27.45
Lab	0.228	0.052	34.88
Residual	0.234	0.055	36.55
Total	0.705	0.149	100.00

Across-lab harmonization (standardized cohort) reduced the overall data variability (i.e., Total variance) compared to the Local protocol as summarized in Table 3: “Total” (model 2). Looking at the proportion of variability explained in each protocol, we found that the variance explained by the variability between laboratories (“Lab” in Table 3) in the Harmonized protocol-standardized cohort (18.67%) decreased by a factor of 3.37 compared to the Local protocol. In addition, this cohort also suggests a more replicable treatment effect across participating labs than the Local protocol as it reduced the variance induced by the drug-by-lab interaction (“DrugTreatment:Lab” term) from approximately 30% to approximately 7% [29.31% to 7.57%].

**Table 3:** Variance components of the across-lab analysis for the Stage 2 Local, Standardized, and Heterogenized protocols (model 2).

Parameter	Local	Standardized	Heterogenized
DrugTreatment:Lab	0.042 (29.31%)	0.006 (7.57%)	0.011 (14.38%)
Lab	0.054 (37.67%)	0.016 (18.67%)	0.024 (30.93%)
Residual	0.048 (33.02%)	0.063 (73.76%)	0.042 (54.69%)
Total	0.144 (100.00%)	0.086 (100.00%)	0.076 (100.00%)

The implementation of the Heterogenized cohort of the Harmonized protocol also reduced the overall variance compared to the Local protocol by a factor of 1.8 but was relatively close to the variance from the Standardized cohort (factor 1.1), respectively (“Total” row in Table 3). Taking a closer look at the proportions of explained variance within protocols, we found that the variance associated with the variability across laboratories in the Heterogenized cohort (31%) was slightly reduced compared to the Local protocol (38%) but not actually larger than the Standardized cohort (19%). Similarly, the interaction of the treatment effect by laboratory in the Heterogenized cohort was reduced compared to the Local protocol by a factor of 3.8, though the Standardized cohort led to an even larger reduction (factor of 8.1).

Furthermore, an extra analysis was performed to explore the individual contribution for each of the 2 environmental factors varied systematically as part of the Heterogenized cohort. The analysis on the light intensity factor revealed that the drug treatment effect across laboratories is not influenced by the light intensity (Tables E and F in S2 Supplementary Stage). Additionally, this factor had no effect on the variability of the measures when included as a random factor in the linear model (Table G in S2 Supplementary Stage).

Similarly, the analysis for the time of testing factor revealed no difference across laboratories for the early versus late time of testing, and this factor did not influence the drug treatment effect (Tables H and I in S2 Supplementary Stage) and also had no influence on the variability of the measure (Table J in S2 Supplementary Stage).

### Stage 3: Local and harmonized (standardized) protocols

**Fig 4:**
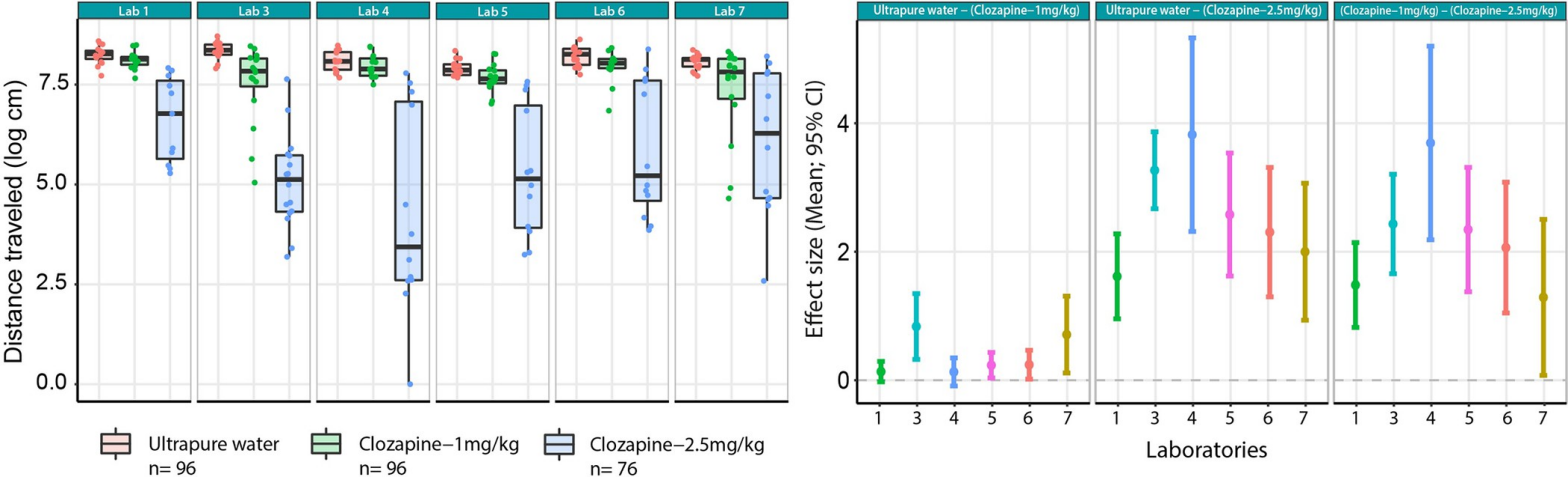
Left graph: Tukey box plots and individual data points across laboratories of the total distance traveled after Clozapine administration (green: 1 mg/kg; blue: 2.5 mg/kg) compared to ultrapure water (red) following the Standardized protocol in stage 3. Right graph: Mean and 95% CI of the treatment effect differences for the Standardized protocol when comparing the control condition to the low dose (left panel), high dose (middle panel), and both doses (right panel) of Clozapine. Data underlying the left panel can be found in https://osf.io/8f6yr/, Stage 3, while data underlying the right panel can be found in Table A in S3 Supplementary Stage.

In this stage, we performed 3 different analyses, each with a different purpose. First, the treatment effect was assessed by comparing the outcome after administration of Clozapine 1 and 2.5 mg/kg, and ultrapure water (Fig 4). Given that this stage was carried out around the contingency of the COVID-19 pandemic, some animal facilities had to stay closed; therefore, Lab 2 was not able to provide data for this stage.

The analysis within each laboratory (model 1) revealed that the Clozapine treatment with 1 mg/kg significantly reduced the distance traveled compared to the ultrapure water for all labs except Labs 1 and 4. However, analysis of the data from these labs revealed a trend with the same direction of the effect (Fig 4, right graph). The highest dose tested, i.e., 2.5 mg/kg Clozapine dose significantly reduced the distance travelled relative to ultrapure water in all sites (Table A in S3 Supplementary Stage).

**Fig 5:**
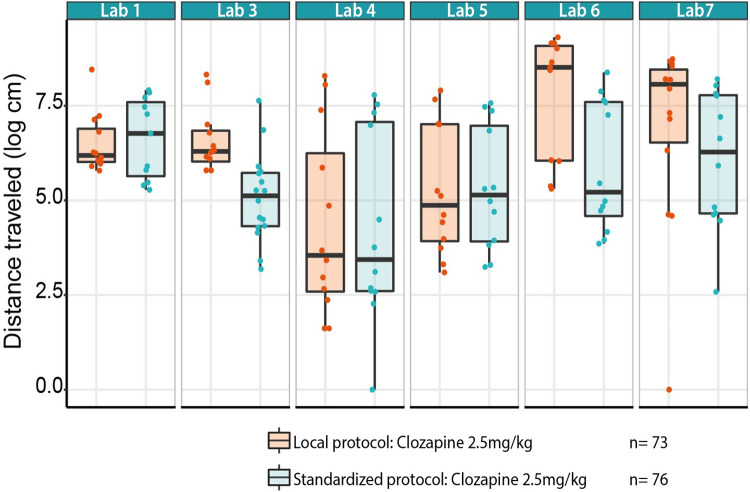
Distance traveled across sites following Clozapine administration (2.5 mg/kg) after implementation of the Local protocol (purple) and Standardized protocol (teal) in stage 3. Data underlying this figure can be found in https://osf.io/8f6yr/, Stage 3.

Secondly, to evaluate the impact of the protocol followed, the between-lab variability was compared when following the standardized protocol with the local protocol, both after 2.5 mg/kg Clozapine (model 1). Both the Local and Standardized protocols showed a similar effect in the distance traveled after Clozapine treatment, although it differed across sites (Fig 5). However, the Standardized protocol reduced the overall variance compared to the Local protocol for this particular treatment (Table 4). In addition, the proportion of the variance explained by the variability across labs when implementing this protocol was reduced by a factor of 2.6 for the 2.5 mg/kg dose of Clozapine (“Lab”; Table 4).

**Table 4:** Variance components for the Local and Standardized protocols in Stage 3 for the 2.5 mg/kg Clozapine treatment (model 1).

Parameter	Local	Standardized
Lab	1.163 (26.38%)	0.436 (12.97%)
Residual	3.247 (73.62%)	2.926 (87.03%)
Total	4.410 (100.00%)	3.362 (100.00%)

Finally, an across-stage comparison (model 3) was made between the control condition of the harmonized protocol (standardized cohort) in stage 3 and the control condition of the same protocol from stage 2. The different stages yielded similar variance components (Table 5, model 1). The variance introduced by using the Standardized protocol in different stages with different vehicles was <1% (Table 6).

**Table 5:** Variance components for the Standardized protocol at its control conditions across stages 2 and 3 (model 1).

Parameter	Standardized (Stage 2)	Standardized (Stage 3)
Lab	0.016 (29.90%)	0.022 (31.01%)
Residual	0.038 (70.10%)	0.048 (68.99%)
Total	0.055 (100.00%)	0.069 (100.00%)

**Table 6:** Comparison across stages 2 and 3 of the standardized protocol for the control condition (model 3).

Parameter	Std. Deviation	Variance Estimate	%
Stage	0.021	<0.001	0.75
Lab	0.128	0.017	26.90
Residual	0.211	0.044	72.35
Total	0.361	0.061	100.00

Lastly, the impact of sex as a blocking factor was explored across laboratories as a fixed effect (Tables B and C in S3 Supplementary Stage). This analysis revealed that sex did not affect the outcome measure as it did not explain the variance of the data.

## Discussion

Overall, our study shows that harmonization of experimental protocols across sites reduced the outcome variability across laboratories compared to site-specific versions of the protocol (i.e., local protocol). Moreover, we found that sex did not affect the results and that illumination of the test arena and time of testing relative to the light–dark cycle were not suitable factors to systematically introduce variation in the results of an open field test in C57BL/6 mice. Regarding the time of testing, we could speculate that the treatment effect had such a strong effect on the outcome variable that there was no room for the time variable to further affect the outcome. Another possible explanation is that this environmental factor does not have a strong influence on the particular outcome tested with the current experimental setup (e.g., the drug and dose used).

The present study showed that between-lab variation is rather large when lab-specific protocols are followed (e.g., local protocol), and, although it was reduced by protocol harmonization, it remained considerable. This corroborates earlier findings [14] that site-specific variation in conditions produces between-lab variability that cannot be neutralized by protocol harmonization across sites. This in turn affects the replicability of study outcomes.

Although the standardized protocol successfully produced replicable results across laboratories, the sensitivity to detect drug treatment effects can still be improved as not all sites found a significant drug treatment effect in stage 3 for the lowest dose (Fig 4; right panel). The choice of the 2 doses tested in stage 3 was based on a literature review performed by one of the partners where the higher dose had a robust effect while the lower dose showed conflicting results. It seems possible that the discrepancy between the sites is due to inherent differences between laboratories that were heightened by the stringent local standardization. It was suggested that a way around this would be to introduce systematic variation within sites, hoping this will account for the variance between sites and test the same drug treatments [17,23].

To test this hypothesis, we introduced systematic variation to the standardized protocol. Contrary to our expectation, this heterogenized cohort did not increase the overall variability, and neither did it decrease the between laboratory variability in outcomes when compared to standardized alone. The overall outcome of the results did not change (i.e., similar drug treatment effects were obtained following the heterogenized and standardized cohorts). Therefore, we could not confirm that diversifying the environmental conditions further reduces the variability across laboratories. The current selection of “heterogenizing” factors was rather limited by the feasibility to diversify them across all labs. Further factors, for example, genotypic variation of the study sample, should be considered for future studies as they may have stronger power to introduce within-study variability than environmental variability as seen in other disciplines [24]. A recent initiative that could prove helpful for identifying heterogenization factors is the Platform for the Exchange of Experimental Research Standards (PEERS) developed to rate the factors and variables most likely to influence experimental outcomes [25].

Moreover, the standardized protocol showed to be robust to the introduction of animals of both sexes in stage 3. Sex did not increase the variability of results across sites compared to the standardized protocol (Table C in S3 Supplementary Stage) and did not account for the variance in the data. In this case, sex may be included without a need to increase the sample size. However, sex should always be included as a biological variable in biomedical research for reasons of inclusion, regardless of its effect on the results [23]. While the harmonization of a standardized protocol across laboratories decreased the overall variability of results compared to when each laboratory followed its own local protocol, the question arises whether these results, although replicable across the participating laboratories, could be further generalized to other laboratories outside the present study. Assuming that the participating laboratories are a representative random sample of laboratories doing phenotyping studies, we could say our results can be extrapolated to other laboratories; however, caution must be taken as the participating labs were all highly interested in data quality and results replicability. This fact might have biased the current sample.

To be able to extrapolate an experimental result to other conditions or populations (i.e., have a broad inference space), the study population has to be representative of the desired target population. Our finding that systematically introducing additional factors (illumination and time of testing in stage 2 and sex in stage 3) did not affect the overall variation shows that diversifying a study population and its environment does not necessarily lead to more “noisy” experimental outcomes but allows to broaden the inference space and increase the external validity of the results and thus their generalizability [26]. This supports diversifying environmental factors that (i) are not tightly linked with the outcome measure or (ii) are not directly involved in the research question as a means to increase the robustness of results. On the other hand, it is necessary to continue exploring the effects of protocol harmonization in results variability since our results suggest that although harmonizing protocols across laboratories reduced between-lab variation, the laboratory factor explains most of the variance, meaning that standardizing is not enough.

## Conclusions

Altogether, we can say that both harmonized (i.e., standardized and heterogenized) open field protocols consistently and significantly reduced the between-lab variability of the behavioral outcome. In addition, the protocols resulted in consistent treatment effects across laboratories that were also replicable within laboratories across the different stages. The replicability of results within and between laboratories in the present study highlights the impact of study-specific variation in between-lab variability, and the importance of transparent and fine-grained reporting of methodologies, and research protocols. It also shows that it is possible to diversify the study sample by incorporating blocking factors like sex or introducing systematic heterogenization of conditions without the need to increase the overall sample size.

## Materials and methods

### General outline

The experiment compared the variability of open field activity in mice after pharmacological treatment across 7 laboratories in Europe, Israel, and the United States, including academic and industry sites. All sites concurrently followed a 3-stage approach wherein different experimental protocols were implemented with the aims to (i) assess the contribution of laboratory-specific (local) protocols to between-lab variability compared to a fully harmonized protocol and (ii) compare a standardized cohort with a heterogenized cohort to assess whether increased diversity enhances external validity, resulting in enhanced replicability.

The selection of the open field test was based on frequent use in the field of biomedical and neuroscience research for the assessment of behavior and specifically for the measurement of locomotor activity levels. Because the purpose of this project was to develop a mechanism for ensuring the concordance of generated data, we decided to focus on one of the simplest yet ubiquitous aspects of behavior, namely locomotion with distance traveled being the primary outcome measure. The ex ante study protocols per site and stage and raw data are publicly available in the OSF repository (DOI: 10.17605/OSF.IO/8F6YR).

### Laboratory sites and ethical statements

All animal procedures were carried out following the regulations of Directive 2010/63/EU or the Association for Assessment and Accreditation of Laboratory Animal Care and following the recommendations of the Guide for the Care and Use of Laboratory Animals. The individual ethical committee for each institution can be found in Table 7.

**Table 7:** Ethical approval committees for each of the laboratories involved.

Laboratory	Ethical approval body
GELIFES (Groningen Institute for Evolutionary Life Sciences, University of Groningen, Groningen, the Netherlands)	Animal Welfare Body of the University of Groningen and the National Central Committee for scientific procedures on animals (CCD)
LMU (Ludwig-Maximilians-Universitaet Muenchen, Muenchen, Germany)	Government of Upper Bavaria (reference number ROB-55.2-2532.Vet_02-18-45)
Orion Pharma (Turku, Finland)	Project Authorization Board in the Regional State Administrative Agency for Southern Finland
PsychoGenics Inc. (New Jersey, USA)	Institutional Animal Care and Used Committee (IACUC #271)
Sylics (Synaptologics BV, Amsterdam, the Netherlands)	Animal Welfare Body of the VU University Amsterdam and the National Central Committee for scientific procedures on animals (CCD)
Teva Pharmaceuticals (Tel Aviv, Israel)	Animal welfare council of the Ministry of Health of Israel (internal committee request #715)
UBERN (Universitaet Bern, Bern, Switzerland)	Cantonal Veterinary Office of the Canton of Bern: License number BE 18/18

In addition, all sites ensured detailed recording of experimental method and procedure according to “The ARRIVE guidelines Animal Research: Reporting In Vivo Experiments” [27] and adhered to the EQIPD key principles for guiding the design, conduct, and analysis of preclinical efficacy and safety research [22].

### Experimental design

#### a. Animals

All experiments were performed with C57BL/6J mice. The details regarding the age, sex, and origin of the animals are summarized in Table 8 as they differed across stages. Likewise, and as part of the 3-stage experimental approach animal numbers, housing, husbandry, and experimental conditions also varied across stages (see Table 8). Note that the animal numbers in Table 8 represent the result of the power calculation; however, some sites included more animals to, for example, even out the total number of animals per group. Thus, the number of observations differs from the one required on Table 8. The animals used in each protocol were experimentally naïve and came from independent batches at all sites. The use of different animal providers among laboratories served as a representation of common differences between laboratories and study populations to test the performance of the different protocols.

**Table 8:** Variables and corresponding values across the different stages and their respective protocol(s) followed by all sites.

FACTOR	STAGE 1	STAGE 2		STAGE 3
	Local	Standardized	Heterogenized	Standardized
***Rearing and housing***				
**Experimental animals**				
Sex	Female and male	Female	Female	Female and male
Strain	C57BL/6J	C57BL/6J	C57BL/6J	C57BL/6J
Age	8–10 weeks	9 weeks	9 weeks	9 weeks
Provider	Variable (In-house, Janvier Lab, Charles River Lab [Germany and France], Envigo [NL and Jerusalem], Jackson Lab)	Variable (In-house, Janvier Lab, Charles River Lab [Germany and France], Envigo [NL and Jerusalem], Jackson Lab)	Variable (In-house, Janvier Lab, Charles River Lab [Germany and France], Envigo [NL and Jerusalem], Jackson Lab)	Variable (In-house, Janvier Lab, Charles River Lab [Germany and France], Envigo [NL and Jerusalem], Jackson Lab)
**Housing**				
Animals per cage	2–5 By sex	3	3	2 By sex
Same sex cage mates	Yes	Yes	Yes	Yes
Cage size	Makrolon I, II L or III	Makrolon III	Makrolon III	Makrolon III
Environmental enrichment type	Variable (e.g., nesting material and shelter or tube and nesting material)	Only 1 type of enrichment (e.g., nesting material or tunnel or shelter)	Only 1 type of enrichment (e.g., nesting material or tunnel or shelter	Only 1 type of enrichment (e.g., nesting material or tunnel or shelter
***Husbandry***				
**Handling method**	Tail or cupped with gloved hands	Tail with gloved hands	Tail with gloved hands	Tail with gloved hands
**Handling frequency**	1–2 times × week	1 time × week	1 time × week	1 time × week
***Behavioral testing***				
**Experimenter gender**	Variable	Female	Female	Female
**Number of handlers**	Multiple	Single/Two^#^ (1 person doing all injections and/or 1 performing the experiment)	Single/Two^#^ (1 person doing all injections and/or 1 performing the experiment)	Single/Two^#^ (1 person doing all injections and/or 1 performing the experiment)
**Acclimation to experimental room**	Variable (0–60 minutes)	60 minutes	60 minutes	60 minutes
**Acquisition method**	IR beam breaks or^#^ Video tracking	IR beam breaks or^#^ Video tracking	IR beam breaks or^#^ Video tracking	IR beam breaks or^#^ Video tracking
**Test arena cleaning method**	Variable	Tap water	Tap water	Tap water
**Drug treatment tested**	Diazepam (Dz) and MK-801 (MK)	MK-801	MK-801	Clozapine (Clz)
**Drug treatment dosage**	Dz: 3 mg/kg MK: 0.2 and 0.3 mg/kg	0.2 mg/kg	0.2 mg/kg	1 and 2.5 mg/kg
**Injection volume and route**	10 mL/kg IP	10 mL/kg IP	10 mL/kg IP	10 mL/kg IP
**Vehicle**	MK: Saline DZ:40% propylene glycol + 10% alcohol + 50% Saline	Saline	Saline	Ultrapure water
**Experimental groups**	5	2	2	3
**Sample sizes**	Saline: 28 (14 Females) MK 0.2 mg/kg: 28 (14F) MK 0.3 mg/kg: 12 (6F) Vehicle Dz: 12 (6F) Dz: 12 (6F)	Saline: 12 MK-801: 12	Saline: 12 MK-801: 12	Ultrapure water: 16 (8F) Clz 1 mg/kg: 16 (8F) Clz 2.5 mg/kg: 12 (6F)
**Treatment assignment**	Variable: random number generator or pick randomly from cage	Block-randomized or balanced across cages*	Block-randomized or balanced across cages*	Block-randomized or balanced across cages*
**Blinded performance and scoring**		Yes	Yes	Yes
**Test duration**	At least 15 minutes	15 minutes	15 minutes	15 minutes
**Test phase**	Light	Light: 4–8 hours after lights ON	Light *Early*: 2–4 hours OR *Late*: 8–10 hours after lights ON	Light: 4–8 hours after lights ON
**Outcome variable**	Distance traveled	Distance traveled	Distance traveled	Distance traveled
**Experimental unit**	Mouse	Cage	Cage	Cage
***Experimental Setup***				
**OF arena shape**	Circular or^#^ square	Square (28 × 28 cm)	Square (28 × 28 cm)	Square (28 × 28 cm)
**OF arena color**	White, gray, or black	White	White	White
**OF arena light intensity**	20–350 Lux	50 Lux	Dim: 20 Lux OR Bright: 80 Lux	50 Lux

*All animals in a cage received the same treatment and were tested in parallel. The reasoning behind this was to avoid social facilitation from “agitated” mice after MK-801 injection influencing control/vehicle mice during the 30-minute wait between the injection and the test; cages and/or animals were block randomized according to the cage location using Blindr tool developed by the VU Amsterdam (https://github.com/jhuebotter/Blindr).

^#^According to the availability at each site; for details, see Table S1 of the Stage 2 protocol available in OSF.

#### b. Design

The readouts of distance traveled in the open field were collected during 3 consecutive stages with around 1 year apart, in 7 different laboratories. In stage 1, all sites performed the study with minimal alignment (strain, age, drug treatment, vehicle, primary outcome measure, and test duration) using their “in-house” standard operation procedures (SOPs) under the local conditions at each site (light intensity, arena size, husbandry conditions, etc.); this is referred to as local protocol and was intended as a baseline measurement of the variability between a “random” sample of laboratories. In stage 2, specific husbandry and experimental conditions were harmonized across laboratories. In addition, besides a standardized cohort of the harmonized protocol, with all factors standardized within laboratories, a heterogenized cohort of the harmonized protocol was used; this cohort aimed to increase within-site variability of the data by systematically varying 2 selected “heterogenization” factors using a 2 × 2 factorial design. Finally, the stage 3 goal was to challenge the sensitivity of the standardized cohort of the harmonized protocol from stage 2 by using the same protocol but with a different drug treatment than previously used (Table 8). The local protocol used in stage 1 was replicated in stages 2 and 3 as a control condition for the harmonized protocols, and to assess the replicability of results obtained with the local protocol across stages within each of the laboratories.

An a priori power analysis (G*Power v3.1.9.2) was performed based on effect sizes estimated from literature [25] and a previous study with the NMDA receptor antagonist MK-801 performed by one of the partners. The study was powered so that each treatment for each sex and each laboratory is treated as a stand-alone test. Alpha was set to 0.05 and power to 0.9 for a *t* test for means-difference for 2 independent means. The calculated effect sizes were considered large. To evaluate the required n numbers in the case of a “medium” effect size, the effect size was halved and corresponding n numbers were again estimated. The required n numbers for “MK-801 0.3 mg/kg” and “3 mg/kg diazepam” were considered too low and were therefore increased to 6. Recommendations of a minimum number of animals to enroll per drug and dose treatment are shown in Table 8. This power calculation was used for the stage 1 protocol. The number of animals used per stage was adapted according to the compounds used and the number of animals available in the animal use licenses.

#### c. Pharmacological compounds

In each of the stages, the spontaneous locomotor activity after acute administration of a compound was compared across treatment groups and/or dosages. While the compounds and dosage used varied across stages (Table 8), the pretreatment time was kept constant with administration 30 minutes before the start of the test. Diazepam (Duchefa Biochemie, BUFA, Roche, Merck, Sigma Aldrich, TEVA) was used in stage 1 together with MK-801 (Sigma Aldrich); the latter was also used in stage 2. Clozapine (Sigma Aldrich cat# C6305) was administered in stage 3. Data from the different drug treatments were compared with the control group that received the respective vehicle (i.e., drug dose = 0 mg/kg). The vehicle was different across treatments according to the compound solubility (see Table 8).

The dose range of each compound was selected according to the goal of each stage as follows. Stage 1: At the localization stage, we aimed for a dosage with a strong effect and a dosage with a medium effect, assuming replicability would be lower with a subtler effect. Doses were based on previous data collected from one of the partners. However, from this, we expected 0.3 mg/kg to have a stronger effect (hence the smaller sample size), but it turned out that it was the other way round. Therefore, we used the smaller dose in stage 2.Stage 2: At stage 2, the focus was on comparing localization with harmonization (primary aim) and standardization with heterogenization (secondary aim); because this increased the number of treatment groups considerably, we limited the study to a single dose against saline control. For the same reason, we limited the study to a single sex (choosing females to minimize the risk of injury and aggression, which is more frequent in males). Thus, stage 2 is a kind of proof-of-principle study to inform stage 3.Stage 3: Similar to stage 1, we wanted a dose with a strong effect and a dose with a weaker effect.

#### d. Protocols

**Local protocol:** A predefined minimum set of requirements were aligned across sites (Table 8). Variables not addressed in these minimum requirements were to be handled according to the SOP of each site. All variables, both aligned and nonaligned, were reported post hoc following stage 1 tests to generate an inventory of the different environmental variables that may have influenced the between-laboratory variability. This protocol represents the most common scenario in preclinical biomedical animal research, where independent studies are standardized within laboratories but conditions and procedures vary between laboratories.

The local protocol was replicated in all stages to test the replicability of results within each laboratory, and as the control protocol to compare the other protocols at each stage. Therefore, the number of animals, drug treatments, and vehicles differed across stages according to Table 8.

**Harmonized protocol–Standardized cohort:** From the local protocol inventories, variables that differed between sites were identified and chosen based on their biological relevance and feasibility to be modified at all sites; these were further harmonized across sites to test whether the variability of effect sizes across sites observed in the local stage could be reduced by controlling for these variables. This cohort aimed to assess how much the lab-specific differences in the standardized protocol contribute to between-lab variation. As indicated in Table 8, this protocol was used in stages 2 and 3; the treatment, vehicle, and treatment dosage differed between stages as well as the inclusion of male mice in stage 3.

**Harmonized protocol–Heterogenized cohort:** This protocol was identical to the standardized cohort, with the exception that 2 factors were systematically varied within sites to account for the variability between sites, namely light intensity in the experimental arena was set to either dim (20 Lux) or bright (80 Lux) and the window time of testing concerning the light–dark phase was varied between early (2 to 4 hours after light on) or late (8 to 10 hours after lights on).

The standardized and heterogenized cohorts of the harmonized protocol were tested in parallel to assess whether simple heterogenization of environmental factors would further reduce the between laboratory variability compared to the local protocol.

### Experimental procedures

#### a. Behavioral assessment

The Open Field (OF) test was used throughout the study to measure the effect of different pharmacological compounds (Table 8) on locomotor activity. Mice were placed in the center of an empty open arena and horizontal activity was recorded for 15 minutes; afterwards, the animal was taken back to its home cage. The outcome measure was the total distance traveled in the OF arena. Details on the open field arenas, recording, and scoring methods are summarized in Table 8.

Animal handling and drug administration, scoring, and analyses were performed blinded to the treatments unless stated otherwise. This means that the person handling and dosing the animals was not aware of the allocation of animals into experimental groups nor about which of the treatments was being administered. Animals were block randomized into groups by various methods (Blindr; random number generator from Mathematica v11, Wolfram; R script provided by one of the partners or developed in-house as part of their Data Management software) except for site 5, which used even distribution of animals into groups. The outcome measure was the total distance traveled for 15 minutes in the Open Field.

There were no predetermined exclusion criteria unless animals presented health issues. However, some sites were able to add animals to, for example, even out the number of animals in each treatment group. Therefore, the raw data has more animals than the ones mentioned in Table 8 for some of the sites.

#### b. Data management

Once data were acquired, each site transferred its raw data and metadata to an Excel structure shared across sites, which was then shared for centralized analysis. Each site was responsible for checking the soundness of the data (i.e., quality check for the video length, accurate scoring, correct group coding, etc.). Averaged data from each treatment group per site across stages can be found in the S1, S2, and S3 Supplementary Stage files.

#### c. Statistical methods

Before the analysis, a log transformation was performed to the outcome variable (i.e., total distance traveled) because data are naturally bounded between 0 and + infinity. The log transformation changes the bound and sets it between −infinity and +infinity, which is more aligned with the assumptions of linear modeling. Moreover, we are sure that the model accounts for those natural bounds when estimating the effects. Otherwise, it could be that some effects have a 95% CI lower bound lower than 0, which would be uninformative given the outcome variable analyzed.

#### Within stages and protocols analysis

To study the lab-to-lab variation by stage and protocol, 2 types of models were used. The first one explored the differences in dosing effects by the laboratory. This analysis reflects a situation where each laboratory would perform the comparisons internally and aims to highlight the variability of estimated differences between laboratories. It was expected that the Harmonized protocol provides more consistent results than the Local protocol. A simple linear regression was fitted to the natural logarithm transform of total distance travelled by laboratory with drug treatment as a unique fixed effect:

$$Y_{id}=\beta_{0}+\beta_{d}\times {dose}_{d}+\varepsilon_{id}$$ (1)

where *Y*_*id*_ is the natural logarithm transform of total distance travelled *i* for drug treatment *d*,

*β*_0_ is the intercept of the model (the expected *Y*_*id*_ for drug treatment *d* of reference),

*β*_*d*_ is the effect of drug treatment *d* on *Y*_*id*_ (the expected change in *Y*_*id*_ when drug treatment *d* is considered), and

*ε*_*id*_ is the random error associated with *Y*_*id*_: *ε*_*id*_~*N*(0, *σ*^2^_*ε*_) where $\sigma_{\varepsilon }^{2}$ is the residual or biological variance.

The drug treatment effects and their contrasts were estimated using the R package emmeans.

Note that for the standardized protocol in stage 3, *Y*_*id*_ is the natural logarithm transform of total distance travelled plus 1 because of some zero values for which the natural logarithm would not be defined. Moreover, also for the standardized protocol in stage 3, huge discrepancies were observed between variances of drug treatment. Hence, 1 variance per drug treatment was modelled instead of 1 pooled variance: $\varepsilon_{id}∼N(0,{\sigma^{2}}_{\varepsilon_{d}})$. This specific model with individual variance per drug treatment was fitted with the R package glmmTMB.

The second model explores the differences in dosing effects overall laboratories accounting for the lab-to-lab variability. It aimed to directly estimate the variance associated with differences between laboratories and assess the percentage of total variance it represented. It was expected that the Harmonized protocol provides lower lab-to-lab variance than the Local protocol while having similar residual variances. A linear mixed model was fitted to the natural logarithm transform of total distance travelled with drug treatment as a unique fixed effect and laboratory as well as the interaction between laboratory and drug treatment as random effects:

$$Y_{idl}=\beta_{0}+\beta_{d}\times {dose}_{d}+b_{l}+d_{dl}+\varepsilon_{idl}$$ (2)

where *Y*_*idl*_ is the natural logarithm transform of total distance travelled *i* for drug treatment *d* and lab *l*,

*β*_0_ is the intercept of the model (the expected *Y*_*idl*_ for drug treatment *d* of reference),

*β*_*d*_ is the effect of drug treatment *d* on *Y*_*idl*_ (the expected change in *Y*_*idl*_ when drug treatment *d* is considered),

*b*_*l*_ = the random intercept of laboratory *l*: $b_{l}∼N(0,\sigma_{b}^{2})$,

*d*_*dl*_ = the random intercept of drug treatment *d* and laboratory *l*: $d_{dl}∼N(0,\sigma_{d}^{2})$, and

*ε*_*idl*_ is the random error associated with *Y*_*idl*_: *ε*_*idl*_~*N*(0,*σ*^2^_*ε*_).

*σ*^2^_*b*_, *σ*^2^_*d*_ and *σ*^2^_*ε*_ are referred as the variance components in this model. In linear mixed model, variance is decomposed in several terms of interest to understand which ones are the main source of variability in the data. In this specific case, *σ*^2^_*b*_ is the lab-to-lab variability, *σ*^2^_*d*_ is the variability in differences between drug doses observed between lab, and *σ*^2^_*ε*_ is the residual or biological variability.

The model was fitted with R package lmer. The drug treatment effects and their contrasts were estimated using the R package emmeans. Note that for the standardized protocol in stage 3, the same modifications were applied as for the simple linear model.

#### Between stages analysis

To study the stage-to-stage variation for 1 protocol, 2 types of approaches were used. The first one compared the within-stage results by stage for common dosing groups. It assesses if effects observed in laboratories and if the variance components are similar from one stage to another. This would indicate that the results are replicable. Local protocols of stages 1 and 2 are compared using the common control and MK-801-0.2 mg/kg groups, whereas Harmonized protocols (standardized cohort) stages 2 and 3 are compared using the common 2.5 mg/kg clozapine treatment. Note that the models presented simplify for the standardized cohort of the Harmonized protocol because there is only 1 dose.

The second model explored the effects over all laboratories accounting for lab-to-lab and stage-to-stage variability. It aimed to estimate the proportion of the total variance attributable to between-stage differences. A linear mixed model was fitted to the natural logarithm transform of the total distance travelled with drug treatment as unique fixed effect and laboratory as well as interaction between laboratory and drug treatment, stage and the interaction between stage and drug treatment as random effects:

$$Y_{idls}=\beta_{0}+\beta_{d}\times {dose}_{d}+b_{l}+d_{dl}+s_{s}+g_{ds}+\varepsilon_{idls}$$ (3)

where *Y*_*idls*_ is the natural logarithm transform of total distance travelled *i* for drug treatment *d*,

lab *l* and stage *s*,

*β*_0_ is the intercept of the model (the expected *Y*_*idls*_ for drug treatment *d* of reference),

*β*_*d*_ is the effect of drug treatment *d* on *Y*_*idls*_ (the expected change in *Y*_*idls*_ when drug treatment *d* is considered),

*b*_*l*_ = the random intercept of laboratory *l*: $b_{l}∼N(0,\sigma_{b}^{2})$,

*d*_*dl*_ = the random intercept of drug treatment *d* and laboratory *l*: $d_{dl}∼N(0,\sigma_{d}^{2})$,

*s*_*s*_ is the random intercept of stage *s*: $s_{s}∼N(0,\sigma_{s}^{2})$,

*g*_*ds*_ is the random intercept of drug treatment *d* and stage *s*: $g_{ds}∼N(0,\sigma_{g}^{2})$, and

*ε*_*idls*_ is the random error associated with *Y*_*idls*_: *ε*_*idls*_~*N*(0, *σ*^2^_*ε*_), where $\sigma_{\varepsilon }^{2}$ is the residual or biological variance.

#### Influence of external factors

Additional models were performed to study the effects of heterogeneous factors introduced for the Harmonized protocol–Heterogenized cohort (the light intensity and the time of testing) in stage 2 and the blocking factor for the Harmonized protocol in stage 3 (sex) on the data. Two approaches were considered, first a by-laboratory analysis, then an across laboratory analysis, both by factor of interest. The models were based on the ones used in the within stages and protocols analysis. Two fixed effects were added each time, the factor and the interaction between the factor and the drug treatment. The models were fitted with R package lmer. Statistical significance of those effects was tested with F tests (Type III) using the R package lmerTest. The different effects and their contrasts were estimated using the R package emmeans.

The boxplots were computed with the raw data that can be found in https://osf.io/8f6yr*/*, while the treatment effect differences are reported in the S1, S2, and S3 Supplementary Stage files. See each figure for specifics. The analysis codes can be found in the OSF repository (DOI: 10.17605/OSF.IO/8F6YR).

## Supporting information

S1 Supplementary Stage Supplementary tables with raw data and statistical results of stage 1. (DOCX)

S2 Supplementary Stage Supplementary tables with raw data and statistical results of stage 2. (DOCX)

S3 Supplementary Stage Supplementary tables with raw data and statistical results of stage 3. (DOCX)

S1 Methods Video analysis settings and husbandry details. (DOCX)

S1 Protocol Supplementary protocol per site for stage 1 with the minimum requirements asked. (DOCX)
