# Supplementary material for: Systematic assessment of the replicability and generalizability of preclinical findings: Impact of protocol harmonization across laboratory sites
Source: PLoS Biol. 2022 Nov 23;20(11):e3001886. doi: 10.1371/journal.pbio.3001886 (PMC9728859; doi:10.1371/journal.pbio.3001886)
Supplement: S1 Supplementary Stage — (DOCX) [file pbio.3001886.s001.docx]

**Table A.** Statistical results of the different MK-801 drug treatments with the local protocol in stage 1.The p-value represent a difference from 0 for a single treatment or a difference between two treatments according to the ‘DrugTreatment’ column.

| Laboratory | DrugTreatment | mean | SE | lower.CL | upper.CL | p-value |
| --- | --- | --- | --- | --- | --- | --- |
| Lab 4 | Saline | 8.03 | 0.044 | 7.95 | 8.12 | <0.0001 |
| Lab 4 | MK-801-0.2 mg/kg | 8.88 | 0.044 | 8.80 | 8.97 | <0.0001 |
| Lab 4 | MK-801-0.3 mg/kg | 8.78 | 0.044 | 8.69 | 8.87 | <0.0001 |
| Lab 4 | Saline - (MK-801-0.2 mg/kg) | -0.85 | 0.062 | -0.97 | -0.73 | <0.0001 |
| Lab 4 | Saline - (MK-801-0.3 mg/kg) | -0.75 | 0.062 | -0.87 | -0.63 | <0.0001 |
| Lab 4 | (MK-801-0.2 mg/kg) - (MK-801-0.3 mg/kg) | 0.10 | 0.062 | -0.02 | 0.23 | 0.0942 |
| Lab 6 | Saline | 9.09 | 0.041 | 9.01 | 9.17 | <0.0001 |
| Lab 6 | MK-801-0.2 mg/kg | 9.28 | 0.041 | 9.19 | 9.36 | <0.0001 |
| Lab 6 | MK-801-0.3 mg/kg | 9.07 | 0.063 | 8.94 | 9.20 | <0.0001 |
| Lab 6 | Saline - (MK-801-0.2 mg/kg) | -0.19 | 0.059 | -0.30 | -0.07 | 0.0022 |
| Lab 6 | Saline - (MK-801-0.3 mg/kg) | 0.02 | 0.076 | -0.13 | 0.17 | 0.8106 |
| Lab 6 | (MK-801-0.2 mg/kg) - (MK-801-0.3 mg/kg) | 0.21 | 0.076 | 0.05 | 0.36 | 0.0085 |
| Lab 2 | Saline | 8.95 | 0.040 | 8.87 | 9.03 | <0.0001 |
| Lab 2 | MK-801-0.2 mg/kg | 9.33 | 0.040 | 9.25 | 9.41 | <0.0001 |
| Lab 2 | MK-801-0.3 mg/kg | 8.98 | 0.062 | 8.86 | 9.11 | <0.0001 |
| Lab 2 | Saline - (MK-801-0.2 mg/kg) | -0.38 | 0.057 | -0.50 | -0.27 | <0.0001 |
| Lab 2 | Saline - (MK-801-0.3 mg/kg) | -0.03 | 0.074 | -0.18 | 0.11 | 0.6455 |
| Lab 2 | (MK-801-0.2 mg/kg) - (MK-801-0.3 mg/kg) | 0.35 | 0.074 | 0.20 | 0.50 | <0.0001 |
| Lab 5 | Saline | 8.23 | 0.049 | 8.14 | 8.33 | <0.0001 |
| Lab 5 | MK-801-0.2 mg/kg | 9.23 | 0.049 | 9.13 | 9.32 | <0.0001 |
| Lab 5 | MK-801-0.3 mg/kg | 9.00 | 0.049 | 8.91 | 9.10 | <0.0001 |
| Lab 5 | Saline - (MK-801-0.2 mg/kg) | -0.99 | 0.069 | -1.13 | -0.86 | <0.0001 |
| Lab 5 | Saline - (MK-801-0.3 mg/kg) | -0.77 | 0.069 | -0.91 | -0.63 | <0.0001 |
| Lab 5 | (MK-801-0.2 mg/kg) - (MK-801-0.3 mg/kg) | 0.22 | 0.069 | 0.09 | 0.36 | 0.0019 |
| Lab 7 | Saline | 8.86 | 0.049 | 8.77 | 8.96 | <0.0001 |
| Lab 7 | MK-801-0.2 mg/kg | 9.19 | 0.047 | 9.09 | 9.28 | <0.0001 |
| Lab 7 | MK-801-0.3 mg/kg | 8.99 | 0.072 | 8.84 | 9.13 | <0.0001 |
| Lab 7 | Saline - (MK-801-0.2 mg/kg) | -0.32 | 0.068 | -0.46 | -0.19 | <0.0001 |
| Lab 7 | Saline - (MK-801-0.3 mg/kg) | -0.12 | 0.087 | -0.30 | 0.05 | 0.1661 |
| Lab 7 | (MK-801-0.2 mg/kg) - (MK-801-0.3 mg/kg) | 0.20 | 0.086 | 0.03 | 0.37 | 0.0233 |
| Lab 1 | Saline | 8.87 | 0.050 | 8.77 | 8.97 | <0.0001 |
| Lab 1 | MK-801-0.2 mg/kg | 9.54 | 0.048 | 9.44 | 9.64 | <0.0001 |
| Lab 1 | MK-801-0.3 mg/kg | 9.53 | 0.074 | 9.38 | 9.68 | <0.0001 |
| Lab 1 | Saline - (MK-801-0.2 mg/kg) | -0.67 | 0.070 | -0.81 | -0.53 | <0.0001 |
| Lab 1 | Saline - (MK-801-0.3 mg/kg) | -0.66 | 0.090 | -0.84 | -0.48 | <0.0001 |
| Lab 1 | (MK-801-0.2 mg/kg) - (MK-801-0.3 mg/kg) | 0.01 | 0.089 | -0.17 | 0.19 | 0.9131 |
| Lab 3 | Saline | 8.78 | 0.051 | 8.68 | 8.88 | <0.0001 |
| Lab 3 | MK-801-0.2 mg/kg | 9.13 | 0.051 | 9.03 | 9.23 | <0.0001 |
| Lab 3 | MK-801-0.3 mg/kg | 9.08 | 0.081 | 8.91 | 9.24 | <0.0001 |
| Lab 3 | Saline - (MK-801-0.2 mg/kg) | -0.35 | 0.072 | -0.50 | -0.21 | <0.0001 |
| Lab 3 | Saline - (MK-801-0.3 mg/kg) | -0.30 | 0.095 | -0.49 | -0.10 | 0.0029 |
| Lab 3 | (MK-801-0.2 mg/kg) - (MK-801-0.3 mg/kg) | 0.06 | 0.095 | -0.13 | 0.25 | 0.5510 |

**Table B.** Raw data for all treatment groups of Stage 1.

| Lab | AnimalID | TestgroupID | NameOfDrug | Dose (mg/kg) | Distance traveled |
| --- | --- | --- | --- | --- | --- |
| Lab 1 | AMW110 | MK 0.2_F | MK-801 | 0.2 | 16324.49384 |
| Lab 1 | AMW111 | MK 0.2_F | MK-801 | 0.2 | 13478.49505 |
| Lab 1 | AMW112 | MK 0.2_F | MK-801 | 0.2 | 14912.39867 |
| Lab 1 | AMW68 | MK 0.2_F | MK-801 | 0.2 | 15358.78503 |
| Lab 1 | AMW77 | MK 0.2_F | MK-801 | 0.2 | 16151.36564 |
| Lab 1 | AMW78 | MK 0.2_F | MK-801 | 0.2 | 18405.76377 |
| Lab 1 | AMW80 | MK 0.2_F | MK-801 | 0.2 | 12724.65393 |
| Lab 1 | AMW81 | MK 0.2_F | MK-801 | 0.2 | 15400.95585 |
| Lab 1 | AMW83 | MK 0.2_F | MK-801 | 0.2 | 13978.59354 |
| Lab 1 | AMW84 | MK 0.2_F | MK-801 | 0.2 | 14121.30012 |
| Lab 1 | AMW85 | MK 0.2_F | MK-801 | 0.2 | 20469.64607 |
| Lab 1 | AMW89 | MK 0.2_F | MK-801 | 0.2 | 17915.0007 |
| Lab 1 | AMW93 | MK 0.2_F | MK-801 | 0.2 | 15513.75349 |
| Lab 1 | AMW97 | MK 0.2_F | MK-801 | 0.2 | 13276.36738 |
| Lab 1 | AMW164 | MK 0.2_M | MK-801 | 0.2 | 15067.082 |
| Lab 1 | AMW166 | MK 0.2_M | MK-801 | 0.2 | 16668.926 |
| Lab 1 | AMW167 | MK 0.2_M | MK-801 | 0.2 | 12293.156 |
| Lab 1 | AMW170 | MK 0.2_M | MK-801 | 0.2 | 7524.687 |
| Lab 1 | AMW171 | MK 0.2_M | MK-801 | 0.2 | 10380.65 |
| Lab 1 | AMW172 | MK 0.2_M | MK-801 | 0.2 | 14183.121 |
| Lab 1 | AMW177 | MK 0.2_M | MK-801 | 0.2 | 15300.368 |
| Lab 1 | AMW185 | MK 0.2_M | MK-801 | 0.2 | 14132.201 |
| Lab 1 | AMW187 | MK 0.2_M | MK-801 | 0.2 | 15977.416 |
| Lab 1 | AMW195 | MK 0.2_M | MK-801 | 0.2 | 8702.236 |
| Lab 1 | AMW198 | MK 0.2_M | MK-801 | 0.2 | 16953.421 |
| Lab 1 | AMW199 | MK 0.2_M | MK-801 | 0.2 | 13006.535 |
| Lab 1 | AMW201 | MK 0.2_M | MK-801 | 0.2 | 9135.541 |
| Lab 1 | AMW204 | MK 0.2_M | MK-801 | 0.2 | 11507.253 |
| Lab 1 | AMW105 | MK 0.3_F | MK-801 | 0.3 | 17772.0835 |
| Lab 1 | AMW73 | MK 0.3_F | MK-801 | 0.3 | 15421.89615 |
| Lab 1 | AMW75 | MK 0.3_F | MK-801 | 0.3 | 17714.9182 |
| Lab 1 | AMW79 | MK 0.3_F | MK-801 | 0.3 | 12454.27852 |
| Lab 1 | AMW86 | MK 0.3_F | MK-801 | 0.3 | 10600.93055 |
| Lab 1 | AMW99 | MK 0.3_F | MK-801 | 0.3 | 18489.59344 |
| Lab 1 | AMW168 | MK 0.3_M | MK-801 | 0.3 | 10781.115 |
| Lab 1 | AMW173 | MK 0.3_M | MK-801 | 0.3 | 10142.058 |
| Lab 1 | AMW184 | MK 0.3_M | MK-801 | 0.3 | 9130.493 |
| Lab 1 | AMW189 | MK 0.3_M | MK-801 | 0.3 | 18584.481 |
| Lab 1 | AMW202 | MK 0.3_M | MK-801 | 0.3 | 12389.254 |
| Lab 1 | AMW210 | MK 0.3_M | MK-801 | 0.3 | 17232.58 |
| Lab 1 | AMW101 | Saline_F | Saline | 0 | 3203.817129 |
| Lab 1 | AMW103 | Saline_F | Saline | 0 | 8180.183647 |
| Lab 1 | AMW104 | Saline_F | Saline | 0 | 8017.27526 |
| Lab 1 | AMW113 | Saline_F | Saline | 0 | 8124.997575 |
| Lab 1 | AMW67 | Saline_F | Saline | 0 | 5337.909277 |
| Lab 1 | AMW69 | Saline_F | Saline | 0 | 7079.506971 |
| Lab 1 | AMW70 | Saline_F | Saline | 0 | 8259.223944 |
| Lab 1 | AMW72 | Saline_F | Saline | 0 | 6351.691891 |
| Lab 1 | AMW74 | Saline_F | Saline | 0 | 7202.036349 |
| Lab 1 | AMW87 | Saline_F | Saline | 0 | 6812.799925 |
| Lab 1 | AMW91 | Saline_F | Saline | 0 | 8297.123503 |
| Lab 1 | AMW95 | Saline_F | Saline | 0 | 7040.925473 |
| Lab 1 | AMW98 | Saline_F | Saline | 0 | 5740.338242 |
| Lab 1 | AMW174 | Saline_M | Saline | 0 | 8030.109 |
| Lab 1 | AMW175 | Saline_M | Saline | 0 | 7164.409 |
| Lab 1 | AMW176 | Saline_M | Saline | 0 | 7574.696 |
| Lab 1 | AMW178 | Saline_M | Saline | 0 | 8214.534 |
| Lab 1 | AMW181 | Saline_M | Saline | 0 | 6635.456 |
| Lab 1 | AMW182 | Saline_M | Saline | 0 | 6974.609 |
| Lab 1 | AMW193 | Saline_M | Saline | 0 | 6837.407 |
| Lab 1 | AMW194 | Saline_M | Saline | 0 | 5448.499 |
| Lab 1 | AMW200 | Saline_M | Saline | 0 | 7527.669 |
| Lab 1 | AMW203 | Saline_M | Saline | 0 | 5290.681 |
| Lab 1 | AMW207 | Saline_M | Saline | 0 | 7980.769 |
| Lab 1 | AMW208 | Saline_M | Saline | 0 | 17906.95622 |
| Lab 1 | AMW209 | Saline_M | Saline | 0 | 7600.973 |
| Lab 1 | AMW100 | Veh DZ_F | Vehicle Diazepam | 0 | 4655.142273 |
| Lab 1 | AMW107 | Veh DZ_F | Vehicle Diazepam | 0 | 8744.308676 |
| Lab 1 | AMW108 | Veh DZ_F | Vehicle Diazepam | 0 | 6529.409526 |
| Lab 1 | AMW71 | Veh DZ_F | Vehicle Diazepam | 0 | 5591.3295 |
| Lab 1 | AMW82 | Veh DZ_F | Vehicle Diazepam | 0 | 6778.575622 |
| Lab 1 | AMW88 | Veh DZ_F | Vehicle Diazepam | 0 | 6429.563946 |
| Lab 1 | AMW183 | Veh DZ_M | Vehicle Diazepam | 0 | 7202.604 |
| Lab 1 | AMW186 | Veh DZ_M | Vehicle Diazepam | 0 | 7526.59 |
| Lab 1 | AMW190 | Veh DZ_M | Vehicle Diazepam | 0 | 7354.27 |
| Lab 1 | AMW191 | Veh DZ_M | Vehicle Diazepam | 0 | 8220.846 |
| Lab 1 | AMW192 | Veh DZ_M | Vehicle Diazepam | 0 | 8454.036 |
| Lab 1 | AMW205 | Veh DZ_M | Vehicle Diazepam | 0 | 10900.067 |
| Lab 2 | PH22159 | DZ00FEMALE | Vehicle Diazepam | 0 | 10225.4 |
| Lab 2 | PH22160 | DZ00FEMALE | Vehicle Diazepam | 0 | 5384.6 |
| Lab 2 | PH22162 | DZ00FEMALE | Vehicle Diazepam | 0 | 6391 |
| Lab 2 | PH22165 | DZ00FEMALE | Vehicle Diazepam | 0 | 5974.9 |
| Lab 2 | PH22168 | DZ00FEMALE | Vehicle Diazepam | 0 | 5878.3 |
| Lab 2 | PH22169 | DZ00FEMALE | Vehicle Diazepam | 0 | 4594.4 |
| Lab 2 | PH22146 | DZ00MALE | Vehicle Diazepam | 0 | 4192.6 |
| Lab 2 | PH22147 | DZ00MALE | Vehicle Diazepam | 0 | 3548.9 |
| Lab 2 | PH22154 | DZ00MALE | Vehicle Diazepam | 0 | 2516.8 |
| Lab 2 | PH22155 | DZ00MALE | Vehicle Diazepam | 0 | 6970.9 |
| Lab 2 | PH22156 | DZ00MALE | Vehicle Diazepam | 0 | 5941.2 |
| Lab 2 | PH22157 | DZ00MALE | Vehicle Diazepam | 0 | 3951.7 |
| Lab 2 | PH22158 | DZ30FEMALE | Diazepam | 0.3 | 2763.8 |
| Lab 2 | PH22161 | DZ30FEMALE | Diazepam | 0.3 | 109 |
| Lab 2 | PH22163 | DZ30FEMALE | Diazepam | 0.3 | 1162.4 |
| Lab 2 | PH22164 | DZ30FEMALE | Diazepam | 0.3 | 3.1 |
| Lab 2 | PH22166 | DZ30FEMALE | Diazepam | 0.3 | 2936.4 |
| Lab 2 | PH22167 | DZ30FEMALE | Diazepam | 0.3 | 829.5 |
| Lab 2 | PH22148 | DZ30MALE | Diazepam | 0.3 | 140 |
| Lab 2 | PH22149 | DZ30MALE | Diazepam | 0.3 | 131.9 |
| Lab 2 | PH22150 | DZ30MALE | Diazepam | 0.3 | 384.1 |
| Lab 2 | PH22151 | DZ30MALE | Diazepam | 0.3 | 44.6 |
| Lab 2 | PH22152 | DZ30MALE | Diazepam | 0.3 | 71.3 |
| Lab 2 | PH22153 | DZ30MALE | Diazepam | 0.3 | 357.4 |
| Lab 2 | PH21918 | MK00FEMALE | Saline | 0 | 6396.3 |
| Lab 2 | PH21919 | MK00FEMALE | Saline | 0 | 8200.4 |
| Lab 2 | PH21920 | MK00FEMALE | Saline | 0 | 9259.1 |
| Lab 2 | PH21923 | MK00FEMALE | Saline | 0 | 8457.6 |
| Lab 2 | PH21925 | MK00FEMALE | Saline | 0 | 9666.1 |
| Lab 2 | PH21928 | MK00FEMALE | Saline | 0 | 5809.4 |
| Lab 2 | PH21930 | MK00FEMALE | Saline | 0 | 7062.7 |
| Lab 2 | PH21934 | MK00FEMALE | Saline | 0 | 7093.7 |
| Lab 2 | PH21937 | MK00FEMALE | Saline | 0 | 8905.6 |
| Lab 2 | PH21940 | MK00FEMALE | Saline | 0 | 6778.1 |
| Lab 2 | PH21942 | MK00FEMALE | Saline | 0 | 7460 |
| Lab 2 | PH21948 | MK00FEMALE | Saline | 0 | 11566.7 |
| Lab 2 | PH21950 | MK00FEMALE | Saline | 0 | 6744.1 |
| Lab 2 | PH21954 | MK00FEMALE | Saline | 0 | 7402.6 |
| Lab 2 | PH21870 | MK00MALE | Saline | 0 | 7577.6 |
| Lab 2 | PH21873 | MK00MALE | Saline | 0 | 6058.1 |
| Lab 2 | PH21875 | MK00MALE | Saline | 0 | 8254.3 |
| Lab 2 | PH21878 | MK00MALE | Saline | 0 | 6322.8 |
| Lab 2 | PH21880 | MK00MALE | Saline | 0 | 9054.4 |
| Lab 2 | PH21885 | MK00MALE | Saline | 0 | 6339.3 |
| Lab 2 | PH21886 | MK00MALE | Saline | 0 | 7106 |
| Lab 2 | PH21892 | MK00MALE | Saline | 0 | 8503.8 |
| Lab 2 | PH21897 | MK00MALE | Saline | 0 | 7109.4 |
| Lab 2 | PH21900 | MK00MALE | Saline | 0 | 8768.9 |
| Lab 2 | PH21902 | MK00MALE | Saline | 0 | 6339.9 |
| Lab 2 | PH21904 | MK00MALE | Saline | 0 | 9142.4 |
| Lab 2 | PH21906 | MK00MALE | Saline | 0 | 9852.4 |
| Lab 2 | PH21907 | MK00MALE | Saline | 0 | 7390.4 |
| Lab 2 | PH21913 | MK02FEMALE | MK-801 | 0.2 | 16218.2 |
| Lab 2 | PH21914 | MK02FEMALE | MK-801 | 0.2 | 11887.1 |
| Lab 2 | PH21915 | MK02FEMALE | MK-801 | 0.2 | 8670.4 |
| Lab 2 | PH21916 | MK02FEMALE | MK-801 | 0.2 | 13146.7 |
| Lab 2 | PH21927 | MK02FEMALE | MK-801 | 0.2 | 9406.9 |
| Lab 2 | PH21933 | MK02FEMALE | MK-801 | 0.2 | 11940 |
| Lab 2 | PH21936 | MK02FEMALE | MK-801 | 0.2 | 12047 |
| Lab 2 | PH21938 | MK02FEMALE | MK-801 | 0.2 | 16364.3 |
| Lab 2 | PH21939 | MK02FEMALE | MK-801 | 0.2 | 15800.9 |
| Lab 2 | PH21943 | MK02FEMALE | MK-801 | 0.2 | 12205.6 |
| Lab 2 | PH21945 | MK02FEMALE | MK-801 | 0.2 | 14940.3 |
| Lab 2 | PH21949 | MK02FEMALE | MK-801 | 0.2 | 11801.8 |
| Lab 2 | PH21955 | MK02FEMALE | MK-801 | 0.2 | 12763.4 |
| Lab 2 | PH21958 | MK02FEMALE | MK-801 | 0.2 | 17020.7 |
| Lab 2 | PH21868 | MK02MALE | MK-801 | 0.2 | 11933.1 |
| Lab 2 | PH21872 | MK02MALE | MK-801 | 0.2 | 6090.8 |
| Lab 2 | PH21874 | MK02MALE | MK-801 | 0.2 | 9470 |
| Lab 2 | PH21879 | MK02MALE | MK-801 | 0.2 | 13153.3 |
| Lab 2 | PH21881 | MK02MALE | MK-801 | 0.2 | 7130 |
| Lab 2 | PH21883 | MK02MALE | MK-801 | 0.2 | 10259.8 |
| Lab 2 | PH21888 | MK02MALE | MK-801 | 0.2 | 11900.4 |
| Lab 2 | PH21889 | MK02MALE | MK-801 | 0.2 | 10732.4 |
| Lab 2 | PH21895 | MK02MALE | MK-801 | 0.2 | 7657.2 |
| Lab 2 | PH21899 | MK02MALE | MK-801 | 0.2 | 11749 |
| Lab 2 | PH21901 | MK02MALE | MK-801 | 0.2 | 8770.7 |
| Lab 2 | PH21903 | MK02MALE | MK-801 | 0.2 | 11311.1 |
| Lab 2 | PH21908 | MK02MALE | MK-801 | 0.2 | 11949.3 |
| Lab 2 | PH21912 | MK02MALE | MK-801 | 0.2 | 9116.7 |
| Lab 2 | PH21921 | MK03FEMALE | MK-801 | 0.3 | 8002 |
| Lab 2 | PH21931 | MK03FEMALE | MK-801 | 0.3 | 5898.4 |
| Lab 2 | PH21935 | MK03FEMALE | MK-801 | 0.3 | 6694.1 |
| Lab 2 | PH21944 | MK03FEMALE | MK-801 | 0.3 | 9683.5 |
| Lab 2 | PH21947 | MK03FEMALE | MK-801 | 0.3 | 9716.1 |
| Lab 2 | PH21951 | MK03FEMALE | MK-801 | 0.3 | 8212.9 |
| Lab 2 | PH21876 | MK03MALE | MK-801 | 0.3 | 9769.8 |
| Lab 2 | PH21887 | MK03MALE | MK-801 | 0.3 | 9765.7 |
| Lab 2 | PH21890 | MK03MALE | MK-801 | 0.3 | 8688.6 |
| Lab 2 | PH21896 | MK03MALE | MK-801 | 0.3 | 8586.1 |
| Lab 2 | PH21909 | MK03MALE | MK-801 | 0.3 | 5356.4 |
| Lab 2 | PH21911 | MK03MALE | MK-801 | 0.3 | 6997.6 |
| Lab 3 | 49 | F / MK 0.2 | MK-801 | 0.2 | 10171.54 |
| Lab 3 | 50 | F / MK 0.2 | MK-801 | 0.2 | 6167.37 |
| Lab 3 | 51 | F / MK 0.2 | MK-801 | 0.2 | 10577.55 |
| Lab 3 | 53 | F / MK 0.2 | MK-801 | 0.2 | 12800.49 |
| Lab 3 | 55 | F / MK 0.2 | MK-801 | 0.2 | 11104.07 |
| Lab 3 | 65 | F / MK 0.2 | MK-801 | 0.2 | 11844.04 |
| Lab 3 | 69 | F / MK 0.2 | MK-801 | 0.2 | 15453.76 |
| Lab 3 | 70 | F / MK 0.2 | MK-801 | 0.2 | 7299.99 |
| Lab 3 | 72 | F / MK 0.2 | MK-801 | 0.2 | 8860.17 |
| Lab 3 | 75 | F / MK 0.2 | MK-801 | 0.2 | 7260.83 |
| Lab 3 | 76 | F / MK 0.2 | MK-801 | 0.2 | 10260.3 |
| Lab 3 | 84 | F / MK 0.2 | MK-801 | 0.2 | 10103.37 |
| Lab 3 | 85 | F / MK 0.2 | MK-801 | 0.2 | 10057.56 |
| Lab 3 | 88 | F / MK 0.2 | MK-801 | 0.2 | 12732.2 |
| Lab 3 | 90 | F / MK 0.2 | MK-801 | 0.2 | 8128.17 |
| Lab 3 | 54 | F / MK 0.3 | MK-801 | 0.3 | 8539.6 |
| Lab 3 | 62 | F / MK 0.3 | MK-801 | 0.3 | 11827.4 |
| Lab 3 | 81 | F / MK 0.3 | MK-801 | 0.3 | 11783.46 |
| Lab 3 | 87 | F / MK 0.3 | MK-801 | 0.3 | 7291.57 |
| Lab 3 | 91 | F / MK 0.3 | MK-801 | 0.3 | 10536.48 |
| Lab 3 | 96 | F / MK 0.3 | MK-801 | 0.3 | 9193.28 |
| Lab 3 | 59 | F / Vehicle (EtOh) | Vehicle Diazepam | 0 | 2362.206 |
| Lab 3 | 63 | F / Vehicle (EtOh) | Vehicle Diazepam | 0 | 5578.52 |
| Lab 3 | 68 | F / Vehicle (EtOh) | Vehicle Diazepam | 0 | 4539.03 |
| Lab 3 | 73 | F / Vehicle (EtOh) | Vehicle Diazepam | 0 | 4447.28 |
| Lab 3 | 78 | F / Vehicle (EtOh) | Vehicle Diazepam | 0 | 2994.941 |
| Lab 3 | 83 | F / Vehicle (EtOh) | Vehicle Diazepam | 0 | 2888.711 |
| Lab 3 | 52 | F / Vehicle (saline) | Saline | 0 | 6720.88 |
| Lab 3 | 56 | F / Vehicle (saline) | Saline | 0 | 16870.73 |
| Lab 3 | 57 | F / Vehicle (saline) | Saline | 0 | 7641.59 |
| Lab 3 | 58 | F / Vehicle (saline) | Saline | 0 | 6996.28 |
| Lab 3 | 61 | F / Vehicle (saline) | Saline | 0 | 5203.021 |
| Lab 3 | 64 | F / Vehicle (saline) | Saline | 0 | 2905.27 |
| Lab 3 | 66 | F / Vehicle (saline) | Saline | 0 | 12030.16 |
| Lab 3 | 67 | F / Vehicle (saline) | Saline | 0 | 6877.02 |
| Lab 3 | 71 | F / Vehicle (saline) | Saline | 0 | 5024.41 |
| Lab 3 | 74 | F / Vehicle (saline) | Saline | 0 | 6293.37 |
| Lab 3 | 80 | F / Vehicle (saline) | Saline | 0 | 7600.57 |
| Lab 3 | 82 | F / Vehicle (saline) | Saline | 0 | 6607.9 |
| Lab 3 | 86 | F / Vehicle (saline) | Saline | 0 | 5341.54 |
| Lab 3 | 92 | F / Vehicle (saline) | Saline | 0 | 11667.05 |
| Lab 3 | 94 | F / Vehicle (saline) | Saline | 0 | 8245.84 |
| Lab 3 | 60 | F/ Diaz 3 | Diazepam | 3 | 2469.8831 |
| Lab 3 | 77 | F/ Diaz 3 | Diazepam | 3 | 2675.442 |
| Lab 3 | 79 | F/ Diaz 3 | Diazepam | 3 | 1314.452 |
| Lab 3 | 89 | F/ Diaz 3 | Diazepam | 3 | 962.6528 |
| Lab 3 | 93 | F/ Diaz 3 | Diazepam | 3 | 1128.265 |
| Lab 3 | 95 | F/ Diaz 3 | Diazepam | 3 | 643.243 |
| Lab 3 | 16 | M / Diaz 3 | Diazepam | 3 | 449.8988 |
| Lab 3 | 21 | M / Diaz 3 | Diazepam | 3 | 658.4101 |
| Lab 3 | 32 | M / Diaz 3 | Diazepam | 3 | 907.783 |
| Lab 3 | 39 | M / Diaz 3 | Diazepam | 3 | 252.913 |
| Lab 3 | 48 | M / Diaz 3 | Diazepam | 3 | 958.173 |
| Lab 3 | 6 | M / Diaz 3 | Diazepam | 3 | 290.4867 |
| Lab 3 | 11 | M / MK 0.2 | MK-801 | 0.2 | 6554.36 |
| Lab 3 | 14 | M / MK 0.2 | MK-801 | 0.2 | 10537.08 |
| Lab 3 | 15 | M / MK 0.2 | MK-801 | 0.2 | 4734.06 |
| Lab 3 | 2 | M / MK 0.2 | MK-801 | 0.2 | 10221.8 |
| Lab 3 | 20 | M / MK 0.2 | MK-801 | 0.2 | 9648.96 |
| Lab 3 | 23 | M / MK 0.2 | MK-801 | 0.2 | 11742.87 |
| Lab 3 | 24 | M / MK 0.2 | MK-801 | 0.2 | 6691.75 |
| Lab 3 | 35 | M / MK 0.2 | MK-801 | 0.2 | 10867.06 |
| Lab 3 | 36 | M / MK 0.2 | MK-801 | 0.2 | 12011.69 |
| Lab 3 | 38 | M / MK 0.2 | MK-801 | 0.2 | 9600.4 |
| Lab 3 | 4 | M / MK 0.2 | MK-801 | 0.2 | 8751.35 |
| Lab 3 | 41 | M / MK 0.2 | MK-801 | 0.2 | 7833.78 |
| Lab 3 | 43 | M / MK 0.2 | MK-801 | 0.2 | 6643.41 |
| Lab 3 | 45 | M / MK 0.2 | MK-801 | 0.2 | 8905.49 |
| Lab 3 | 47 | M / MK 0.2 | MK-801 | 0.2 | 8691.25 |
| Lab 3 | 1 | M / Veh (saline) | Saline | 0 | 5880.93 |
| Lab 3 | 10 | M / Veh (saline) | Saline | 0 | 6202.79 |
| Lab 3 | 12 | M / Veh (saline) | Saline | 0 | 3563.526 |
| Lab 3 | 13 | M / Veh (saline) | Saline | 0 | 5787.58 |
| Lab 3 | 19 | M / Veh (saline) | Saline | 0 | 6747.13 |
| Lab 3 | 22 | M / Veh (saline) | Saline | 0 | 6506.15 |
| Lab 3 | 28 | M / Veh (saline) | Saline | 0 | 6338.74 |
| Lab 3 | 30 | M / Veh (saline) | Saline | 0 | 6298.19 |
| Lab 3 | 33 | M / Veh (saline) | Saline | 0 | 6814.48 |
| Lab 3 | 37 | M / Veh (saline) | Saline | 0 | 7103.86 |
| Lab 3 | 40 | M / Veh (saline) | Saline | 0 | 6349.921 |
| Lab 3 | 42 | M / Veh (saline) | Saline | 0 | 6341.25 |
| Lab 3 | 46 | M / Veh (saline) | Saline | 0 | 5824.526 |
| Lab 3 | 5 | M / Veh (saline) | Saline | 0 | 5868.32 |
| Lab 3 | 9 | M / Veh (saline) | Saline | 0 | 4646.96 |
| Lab 3 | 17 | M Veh (EtOH) | Vehicle Diazepam | 0 | 5489.43 |
| Lab 3 | 18 | M Veh (EtOH) | Vehicle Diazepam | 0 | 3702.01 |
| Lab 3 | 26 | M Veh (EtOH) | Vehicle Diazepam | 0 | 3960.72 |
| Lab 3 | 29 | M Veh (EtOH) | Vehicle Diazepam | 0 | 2363.14 |
| Lab 3 | 31 | M Veh (EtOH) | Vehicle Diazepam | 0 | 2871.069 |
| Lab 3 | 34 | M Veh (EtOH) | Vehicle Diazepam | 0 | 3513.839 |
| Lab 3 | 25 | M/ MK 0.3 | MK-801 | 0.3 | 7351.37 |
| Lab 3 | 27 | M/ MK 0.3 | MK-801 | 0.3 | 7421.36 |
| Lab 3 | 3 | M/ MK 0.3 | MK-801 | 0.3 | 8799.53 |
| Lab 3 | 44 | M/ MK 0.3 | MK-801 | 0.3 | 7445.83 |
| Lab 3 | 7 | M/ MK 0.3 | MK-801 | 0.3 | 9232.07 |
| Lab 3 | 8 | M/ MK 0.3 | MK-801 | 0.3 | 7168.98 |
| Lab 4 | C57BL60260.04 | DZ_F | Diazepam | 3 | 494.9773 |
| Lab 4 | C57BL60260.05 | DZ_F | Diazepam | 3 | 1495.5483 |
| Lab 4 | C57BL60261.04 | DZ_F | Diazepam | 3 | 240.145 |
| Lab 4 | C57BL60261.05 | DZ_F | Diazepam | 3 | 0 |
| Lab 4 | C57BL60270.04 | DZ_F | Diazepam | 3 | 911.2519 |
| Lab 4 | C57BL60270.05 | DZ_F | Diazepam | 3 | 13.1773 |
| Lab 4 | C57BL60275.04 | DZ_M | Diazepam | 3 | 181.7751 |
| Lab 4 | C57BL60275.05 | DZ_M | Diazepam | 3 | 4.6283 |
| Lab 4 | C57BL60276.01 | DZ_M | Diazepam | 3 | 1177.1274 |
| Lab 4 | C57BL60276.02 | DZ_M | Diazepam | 3 | 47.7364 |
| Lab 4 | C57BL60277.04 | DZ_M | Diazepam | 3 | 24.1284 |
| Lab 4 | C57BL60277.05 | DZ_M | Diazepam | 3 | 0 |
| Lab 4 | C57BL60259.01 | MK 0.2_F | MK-801 | 0.2 | 8439.9479 |
| Lab 4 | C57BL60259.02 | MK 0.2_F | MK-801 | 0.2 | 4987.8738 |
| Lab 4 | C57BL60259.03 | MK 0.2_F | MK-801 | 0.2 | 7742.6307 |
| Lab 4 | C57BL60259.04 | MK 0.2_F | MK-801 | 0.2 | 7057.7825 |
| Lab 4 | C57BL60259.05 | MK 0.2_F | MK-801 | 0.2 | 7558.2088 |
| Lab 4 | C57BL60262.01 | MK 0.2_F | MK-801 | 0.2 | 6979.8062 |
| Lab 4 | C57BL60262.02 | MK 0.2_F | MK-801 | 0.2 | 6165.2802 |
| Lab 4 | C57BL60262.03 | MK 0.2_F | MK-801 | 0.2 | 8183.6173 |
| Lab 4 | C57BL60262.04 | MK 0.2_F | MK-801 | 0.2 | 6898.1569 |
| Lab 4 | C57BL60262.05 | MK 0.2_F | MK-801 | 0.2 | 8175.517 |
| Lab 4 | C57BL60266.02 | MK 0.2_F | MK-801 | 0.2 | 6167.7602 |
| Lab 4 | C57BL60266.03 | MK 0.2_F | MK-801 | 0.2 | 7975.6504 |
| Lab 4 | C57BL60266.04 | MK 0.2_F | MK-801 | 0.2 | 11557.045 |
| Lab 4 | C57BL60266.05 | MK 0.2_F | MK-801 | 0.2 | 7370.4718 |
| Lab 4 | C57BL60282.01 | MK 0.2_M | MK-801 | 0.2 | 7487.195 |
| Lab 4 | C57BL60282.02 | MK 0.2_M | MK-801 | 0.2 | 5346.475 |
| Lab 4 | C57BL60282.03 | MK 0.2_M | MK-801 | 0.2 | 8476.7334 |
| Lab 4 | C57BL60282.05 | MK 0.2_M | MK-801 | 0.2 | 7697.6169 |
| Lab 4 | C57BL60283.01 | MK 0.2_M | MK-801 | 0.2 | 7523.1499 |
| Lab 4 | C57BL60283.02 | MK 0.2_M | MK-801 | 0.2 | 8275.4974 |
| Lab 4 | C57BL60283.03 | MK 0.2_M | MK-801 | 0.2 | 4475.3503 |
| Lab 4 | C57BL60283.04 | MK 0.2_M | MK-801 | 0.2 | 6581.4652 |
| Lab 4 | C57BL60283.05 | MK 0.2_M | MK-801 | 0.2 | 7056.2236 |
| Lab 4 | C57BL60284.01 | MK 0.2_M | MK-801 | 0.2 | 6329.5941 |
| Lab 4 | C57BL60284.02 | MK 0.2_M | MK-801 | 0.2 | 8049.587 |
| Lab 4 | C57BL60284.03 | MK 0.2_M | MK-801 | 0.2 | 6451.5016 |
| Lab 4 | C57BL60284.04 | MK 0.2_M | MK-801 | 0.2 | 7374.0695 |
| Lab 4 | C57BL60284.05 | MK 0.2_M | MK-801 | 0.2 | 9102.8028 |
| Lab 4 | C57BL60263.01 | MK 0.3_F | MK-801 | 0.3 | 9174.1918 |
| Lab 4 | C57BL60263.02 | MK 0.3_F | MK-801 | 0.3 | 5896.3173 |
| Lab 4 | C57BL60263.03 | MK 0.3_F | MK-801 | 0.3 | 6700.712 |
| Lab 4 | C57BL60263.04 | MK 0.3_F | MK-801 | 0.3 | 9843.2574 |
| Lab 4 | C57BL60263.05 | MK 0.3_F | MK-801 | 0.3 | 7101.7413 |
| Lab 4 | C57BL60271.01 | MK 0.3_F | MK-801 | 0.3 | 3433.2957 |
| Lab 4 | C57BL60271.02 | MK 0.3_F | MK-801 | 0.3 | 6841.9369 |
| Lab 4 | C57BL60271.03 | MK 0.3_F | MK-801 | 0.3 | 7121.092 |
| Lab 4 | C57BL60271.05 | MK 0.3_F | MK-801 | 0.3 | 9477.074 |
| Lab 4 | C57BL60272.01 | MK 0.3_F | MK-801 | 0.3 | 4751.4027 |
| Lab 4 | C57BL60272.02 | MK 0.3_F | MK-801 | 0.3 | 6186.3299 |
| Lab 4 | C57BL60272.03 | MK 0.3_F | MK-801 | 0.3 | 9264.1265 |
| Lab 4 | C57BL60272.04 | MK 0.3_F | MK-801 | 0.3 | 9721.2157 |
| Lab 4 | C57BL60272.05 | MK 0.3_F | MK-801 | 0.3 | 5755.849 |
| Lab 4 | C57BL60280.01 | MK 0.3_M | MK-801 | 0.3 | 5866.2826 |
| Lab 4 | C57BL60280.02 | MK 0.3_M | MK-801 | 0.3 | 8261.0142 |
| Lab 4 | C57BL60280.03 | MK 0.3_M | MK-801 | 0.3 | 5137.9846 |
| Lab 4 | C57BL60280.05 | MK 0.3_M | MK-801 | 0.3 | 7688.6723 |
| Lab 4 | C57BL60281.01 | MK 0.3_M | MK-801 | 0.3 | 6304.4711 |
| Lab 4 | C57BL60281.02 | MK 0.3_M | MK-801 | 0.3 | 4200.6253 |
| Lab 4 | C57BL60281.03 | MK 0.3_M | MK-801 | 0.3 | 8127.7698 |
| Lab 4 | C57BL60281.04 | MK 0.3_M | MK-801 | 0.3 | 5135.4849 |
| Lab 4 | C57BL60281.05 | MK 0.3_M | MK-801 | 0.3 | 7682.8738 |
| Lab 4 | C57BL60286.01 | MK 0.3_M | MK-801 | 0.3 | 6422.0953 |
| Lab 4 | C57BL60286.02 | MK 0.3_M | MK-801 | 0.3 | 6722.9853 |
| Lab 4 | C57BL60286.03 | MK 0.3_M | MK-801 | 0.3 | 5223.1946 |
| Lab 4 | C57BL60286.04 | MK 0.3_M | MK-801 | 0.3 | 5830.0747 |
| Lab 4 | C57BL60286.05 | MK 0.3_M | MK-801 | 0.3 | 4451.8059 |
| Lab 4 | C57BL60264.01 | Saline_F | Saline | 0 | 3000.6958 |
| Lab 4 | C57BL60264.02 | Saline_F | Saline | 0 | 2716.1216 |
| Lab 4 | C57BL60264.03 | Saline_F | Saline | 0 | 3697.3293 |
| Lab 4 | C57BL60264.04 | Saline_F | Saline | 0 | 3780.8002 |
| Lab 4 | C57BL60264.05 | Saline_F | Saline | 0 | 3159.7318 |
| Lab 4 | C57BL60265.01 | Saline_F | Saline | 0 | 2385.2651 |
| Lab 4 | C57BL60265.02 | Saline_F | Saline | 0 | 3023.2289 |
| Lab 4 | C57BL60265.03 | Saline_F | Saline | 0 | 4704.5971 |
| Lab 4 | C57BL60265.04 | Saline_F | Saline | 0 | 4874.2905 |
| Lab 4 | C57BL60265.05 | Saline_F | Saline | 0 | 3473.1376 |
| Lab 4 | C57BL60269.02 | Saline_F | Saline | 0 | 3435.5615 |
| Lab 4 | C57BL60269.03 | Saline_F | Saline | 0 | 4006.7052 |
| Lab 4 | C57BL60269.04 | Saline_F | Saline | 0 | 2852.567 |
| Lab 4 | C57BL60269.05 | Saline_F | Saline | 0 | 4081.4961 |
| Lab 4 | C57BL60273.02 | Saline_M | Saline | 0 | 2083.4233 |
| Lab 4 | C57BL60273.03 | Saline_M | Saline | 0 | 2000.1169 |
| Lab 4 | C57BL60273.04 | Saline_M | Saline | 0 | 3103.8545 |
| Lab 4 | C57BL60273.05 | Saline_M | Saline | 0 | 2516.6602 |
| Lab 4 | C57BL60274.01 | Saline_M | Saline | 0 | 3264.4608 |
| Lab 4 | C57BL60274.02 | Saline_M | Saline | 0 | 3145.6772 |
| Lab 4 | C57BL60274.03 | Saline_M | Saline | 0 | 3208.6597 |
| Lab 4 | C57BL60274.04 | Saline_M | Saline | 0 | 2995.3035 |
| Lab 4 | C57BL60274.05 | Saline_M | Saline | 0 | 2753.0665 |
| Lab 4 | C57BL60278.01 | Saline_M | Saline | 0 | 3296.1236 |
| Lab 4 | C57BL60278.02 | Saline_M | Saline | 0 | 2291.1895 |
| Lab 4 | C57BL60278.03 | Saline_M | Saline | 0 | 2228.8296 |
| Lab 4 | C57BL60278.04 | Saline_M | Saline | 0 | 3651.8188 |
| Lab 4 | C57BL60278.05 | Saline_M | Saline | 0 | 2590.3611 |
| Lab 4 | C57BL60258.04 | Veh DZ_F | Vehicle Diazepam | 0 | 1714.3747 |
| Lab 4 | C57BL60258.05 | Veh DZ_F | Vehicle Diazepam | 0 | 709.3294 |
| Lab 4 | C57BL60267.04 | Veh DZ_F | Vehicle Diazepam | 0 | 1586.8653 |
| Lab 4 | C57BL60267.05 | Veh DZ_F | Vehicle Diazepam | 0 | 1912.8975 |
| Lab 4 | C57BL60268.04 | Veh DZ_F | Vehicle Diazepam | 0 | 2094.6116 |
| Lab 4 | C57BL60268.05 | Veh DZ_F | Vehicle Diazepam | 0 | 2138.4403 |
| Lab 4 | C57BL60279.01 | Veh DZ_M | Vehicle Diazepam | 0 | 1451.7118 |
| Lab 4 | C57BL60279.02 | Veh DZ_M | Vehicle Diazepam | 0 | 1303.5727 |
| Lab 4 | C57BL60285.04 | Veh DZ_M | Vehicle Diazepam | 0 | 841.4899 |
| Lab 4 | C57BL60285.05 | Veh DZ_M | Vehicle Diazepam | 0 | 1650.7354 |
| Lab 4 | C57BL60287.04 | Veh DZ_M | Vehicle Diazepam | 0 | 2715.4602 |
| Lab 4 | C57BL60287.05 | Veh DZ_M | Vehicle Diazepam | 0 | 1545.3805 |
| Lab 5 | 10m | 1 | Saline | 0 | 4413.06 |
| Lab 5 | 13m | 1 | Saline | 0 | 3976.8 |
| Lab 5 | 16m | 1 | Saline | 0 | 5068.21 |
| Lab 5 | 19m | 1 | Saline | 0 | 3830.94 |
| Lab 5 | 1m | 1 | Saline | 0 | 3882.28 |
| Lab 5 | 22m | 1 | Saline | 0 | 2611.93 |
| Lab 5 | 41m | 1 | Saline | 0 | 3546.99 |
| Lab 5 | 44m | 1 | Saline | 0 | 4885.44 |
| Lab 5 | 4m | 1 | Saline | 0 | 4542.49 |
| Lab 5 | 7m | 1 | Saline | 0 | 2776.13 |
| Lab 5 | 11m | 2 | MK-801 | 0.2 | 12301.4 |
| Lab 5 | 14m | 2 | MK-801 | 0.2 | 11075.1 |
| Lab 5 | 17m | 2 | MK-801 | 0.2 | 9348.28 |
| Lab 5 | 20m | 2 | MK-801 | 0.2 | 10171.9 |
| Lab 5 | 23m | 2 | MK-801 | 0.2 | 10129.8 |
| Lab 5 | 2m | 2 | MK-801 | 0.2 | 10839.6 |
| Lab 5 | 42m | 2 | MK-801 | 0.2 | 9263.06 |
| Lab 5 | 45m | 2 | MK-801 | 0.2 | 11879.5 |
| Lab 5 | 5m | 2 | MK-801 | 0.2 | 10853.9 |
| Lab 5 | 8m | 2 | MK-801 | 0.2 | 10482.3 |
| Lab 5 | 12m | 3 | MK-801 | 0.3 | 7484.02 |
| Lab 5 | 15m | 3 | MK-801 | 0.3 | 7662.73 |
| Lab 5 | 18m | 3 | MK-801 | 0.3 | 9288.94 |
| Lab 5 | 21m | 3 | MK-801 | 0.3 | 9628.16 |
| Lab 5 | 24m | 3 | MK-801 | 0.3 | 9297.27 |
| Lab 5 | 3m | 3 | MK-801 | 0.3 | 5875.71 |
| Lab 5 | 43m | 3 | MK-801 | 0.3 | 8920.93 |
| Lab 5 | 46m | 3 | MK-801 | 0.3 | 8624.43 |
| Lab 5 | 6m | 3 | MK-801 | 0.3 | 7279.75 |
| Lab 5 | 9m | 3 | MK-801 | 0.3 | 7874.18 |
| Lab 5 | 25m | 4 | Vehicle Diazepam | 0 | 1110.14 |
| Lab 5 | 27m | 4 | Vehicle Diazepam | 0 | 809.098 |
| Lab 5 | 29m | 4 | Vehicle Diazepam | 0 | 2491.84 |
| Lab 5 | 31m | 4 | Vehicle Diazepam | 0 | 1700.63 |
| Lab 5 | 26m | 5 | Diazepam | 3 | 83.9887 |
| Lab 5 | 28m | 5 | Diazepam | 3 | 64.2337 |
| Lab 5 | 30m | 5 | Diazepam | 3 | 57.7845 |
| Lab 5 | 32m | 5 | Diazepam | 3 | 68.2927 |
| Lab 5 | 12f | 6 | Saline | 0 | 3988.45 |
| Lab 5 | 15f | 6 | Saline | 0 | 3560.71 |
| Lab 5 | 18f | 6 | Saline | 0 | 4013.1 |
| Lab 5 | 21f | 6 | Saline | 0 | 5097.44 |
| Lab 5 | 24f | 6 | Saline | 0 | 3883.64 |
| Lab 5 | 3f | 6 | Saline | 0 | 2939.84 |
| Lab 5 | 41f | 6 | Saline | 0 | 3932.65 |
| Lab 5 | 44f | 6 | Saline | 0 | 2823.59 |
| Lab 5 | 6f | 6 | Saline | 0 | 3745.68 |
| Lab 5 | 9f | 6 | Saline | 0 | 3189.99 |
| Lab 5 | 10f | 7 | MK-801 | 0.2 | 9203.48 |
| Lab 5 | 13f | 7 | MK-801 | 0.2 | 10222.2 |
| Lab 5 | 16f | 7 | MK-801 | 0.2 | 10208 |
| Lab 5 | 19f | 7 | MK-801 | 0.2 | 9933.49 |
| Lab 5 | 1f | 7 | MK-801 | 0.2 | 8439.62 |
| Lab 5 | 22f | 7 | MK-801 | 0.2 | 11792.4 |
| Lab 5 | 42f | 7 | MK-801 | 0.2 | 11656.4 |
| Lab 5 | 47f | 7 | MK-801 | 0.2 | 13108.4 |
| Lab 5 | 4f | 7 | MK-801 | 0.2 | 11116.7 |
| Lab 5 | 7f | 7 | MK-801 | 0.2 | 4895.24 |
| Lab 5 | 11f | 8 | MK-801 | 0.3 | 5403.12 |
| Lab 5 | 14f | 8 | MK-801 | 0.3 | 7810.19 |
| Lab 5 | 17f | 8 | MK-801 | 0.3 | 8666.03 |
| Lab 5 | 20f | 8 | MK-801 | 0.3 | 11372.1 |
| Lab 5 | 23f | 8 | MK-801 | 0.3 | 10581.1 |
| Lab 5 | 2f | 8 | MK-801 | 0.3 | 8846.3 |
| Lab 5 | 43f | 8 | MK-801 | 0.3 | 11034.1 |
| Lab 5 | 48f | 8 | MK-801 | 0.3 | 8202.2 |
| Lab 5 | 5f | 8 | MK-801 | 0.3 | 9112.16 |
| Lab 5 | 8f | 8 | MK-801 | 0.3 | 3970.78 |
| Lab 5 | 45f | 9 | Vehicle Diazepam | NA | 1861.78 |
| Lab 5 | 49f | 9 | Vehicle Diazepam | NA | 1121.42 |
| Lab 5 | 46f | 10 | Diazepam | 3 | 47.6524 |
| Lab 5 | 50f | 10 | Diazepam | 3 | 65.9213 |
| Lab 6 | 33820 | Diazepam_F | Diazepam | 3 | 79.2684 |
| Lab 6 | 33866 | Diazepam_F | Diazepam | 3 | 663.4 |
| Lab 6 | 33876 | Diazepam_F | Diazepam | 3 | 1013.97 |
| Lab 6 | 34053 | Diazepam_F | Diazepam | 3 | 4595.53 |
| Lab 6 | 34059 | Diazepam_F | Diazepam | 3 | 2494.6 |
| Lab 6 | 34068 | Diazepam_F | Diazepam | 3 | 92.5207 |
| Lab 6 | 33838 | Diazepam_M | Diazepam | 3 | 103.123 |
| Lab 6 | 33846 | Diazepam_M | Diazepam | 3 | 359.679 |
| Lab 6 | 33881 | Diazepam_M | Diazepam | 3 | 195.62 |
| Lab 6 | 34065 | Diazepam_M | Diazepam | 3 | 99.5892 |
| Lab 6 | 34076 | Diazepam_M | Diazepam | 3 | 275.67 |
| Lab 6 | 34085 | Diazepam_M | Diazepam | 3 | 61.5095 |
| Lab 6 | 33819 | MK 0.2_F | MK-801 | 0.2 | 12392.3 |
| Lab 6 | 33822 | MK 0.2_F | MK-801 | 0.2 | 12164.1 |
| Lab 6 | 33843 | MK 0.2_F | MK-801 | 0.2 | 10535.9 |
| Lab 6 | 33853 | MK 0.2_F | MK-801 | 0.2 | 15190.2 |
| Lab 6 | 33872 | MK 0.2_F | MK-801 | 0.2 | 13051.1 |
| Lab 6 | 33874 | MK 0.2_F | MK-801 | 0.2 | 7012.03 |
| Lab 6 | 33882 | MK 0.2_F | MK-801 | 0.2 | 9816.14 |
| Lab 6 | 34056 | MK 0.2_F | MK-801 | 0.2 | 15010.9 |
| Lab 6 | 34062 | MK 0.2_F | MK-801 | 0.2 | 10125.7 |
| Lab 6 | 34080 | MK 0.2_F | MK-801 | 0.2 | 11453.4 |
| Lab 6 | 34086 | MK 0.2_F | MK-801 | 0.2 | 11984.8 |
| Lab 6 | 34088 | MK 0.2_F | MK-801 | 0.2 | 7708.44 |
| Lab 6 | 34101 | MK 0.2_F | MK-801 | 0.2 | 13726.5 |
| Lab 6 | 34106 | MK 0.2_F | MK-801 | 0.2 | 8368.6 |
| Lab 6 | 33828 | MK 0.2_M | MK-801 | 0.2 | 11181.5 |
| Lab 6 | 33836 | MK 0.2_M | MK-801 | 0.2 | 10890.2 |
| Lab 6 | 33839 | MK 0.2_M | MK-801 | 0.2 | 13336.2 |
| Lab 6 | 33845 | MK 0.2_M | MK-801 | 0.2 | 7818.03 |
| Lab 6 | 33847 | MK 0.2_M | MK-801 | 0.2 | 8142.81 |
| Lab 6 | 33854 | MK 0.2_M | MK-801 | 0.2 | 10570.7 |
| Lab 6 | 33868 | MK 0.2_M | MK-801 | 0.2 | 10048.5 |
| Lab 6 | 33877 | MK 0.2_M | MK-801 | 0.2 | 10425.6 |
| Lab 6 | 33879 | MK 0.2_M | MK-801 | 0.2 | 10386.2 |
| Lab 6 | 34060 | MK 0.2_M | MK-801 | 0.2 | 9869.15 |
| Lab 6 | 34064 | MK 0.2_M | MK-801 | 0.2 | 9915.79 |
| Lab 6 | 34072 | MK 0.2_M | MK-801 | 0.2 | 12905.9 |
| Lab 6 | 34082 | MK 0.2_M | MK-801 | 0.2 | 8629.81 |
| Lab 6 | 34104 | MK 0.2_M | MK-801 | 0.2 | 12045.5 |
| Lab 6 | 33818 | MK 0.3_F | MK-801 | 0.3 | 7191.44 |
| Lab 6 | 33840 | MK 0.3_F | MK-801 | 0.3 | 6354.15 |
| Lab 6 | 33844 | MK 0.3_F | MK-801 | 0.3 | 9548.87 |
| Lab 6 | 33871 | MK 0.3_F | MK-801 | 0.3 | 13603.3 |
| Lab 6 | 34058 | MK 0.3_F | MK-801 | 0.3 | 15075 |
| Lab 6 | 34109 | MK 0.3_F | MK-801 | 0.3 | 8603.53 |
| Lab 6 | 33829 | MK 0.3_M | MK-801 | 0.3 | 6059.78 |
| Lab 6 | 33837 | MK 0.3_M | MK-801 | 0.3 | 10920.2 |
| Lab 6 | 33856 | MK 0.3_M | MK-801 | 0.3 | 5782.99 |
| Lab 6 | 34063 | MK 0.3_M | MK-801 | 0.3 | 8640.84 |
| Lab 6 | 34070 | MK 0.3_M | MK-801 | 0.3 | 8628.09 |
| Lab 6 | 34073 | MK 0.3_M | MK-801 | 0.3 | 8528.59 |
| Lab 6 | 33821 | Saline_F | Saline | 0 | 9086.93 |
| Lab 6 | 33823 | Saline_F | Saline | 0 | 10299.3 |
| Lab 6 | 33841 | Saline_F | Saline | 0 | 6967.42 |
| Lab 6 | 33842 | Saline_F | Saline | 0 | 7700.73 |
| Lab 6 | 33867 | Saline_F | Saline | 0 | 11444.7 |
| Lab 6 | 33883 | Saline_F | Saline | 0 | 7819.51 |
| Lab 6 | 34052 | Saline_F | Saline | 0 | 10710.2 |
| Lab 6 | 34057 | Saline_F | Saline | 0 | 12905.9 |
| Lab 6 | 34067 | Saline_F | Saline | 0 | 7714.07 |
| Lab 6 | 34074 | Saline_F | Saline | 0 | 8404.19 |
| Lab 6 | 34079 | Saline_F | Saline | 0 | 8345.12 |
| Lab 6 | 34081 | Saline_F | Saline | 0 | 9642.76 |
| Lab 6 | 34089 | Saline_F | Saline | 0 | 8628.09 |
| Lab 6 | 34107 | Saline_F | Saline | 0 | 7410.62 |
| Lab 6 | 33827 | Saline_M | Saline | 0 | 10613.7 |
| Lab 6 | 33855 | Saline_M | Saline | 0 | 7845.71 |
| Lab 6 | 33857 | Saline_M | Saline | 0 | 9371.73 |
| Lab 6 | 33869 | Saline_M | Saline | 0 | 9304.36 |
| Lab 6 | 33878 | Saline_M | Saline | 0 | 9378.64 |
| Lab 6 | 33880 | Saline_M | Saline | 0 | 12803.6 |
| Lab 6 | 34054 | Saline_M | Saline | 0 | 8922.26 |
| Lab 6 | 34055 | Saline_M | Saline | 0 | 8206.7 |
| Lab 6 | 34061 | Saline_M | Saline | 0 | 5872.37 |
| Lab 6 | 34069 | Saline_M | Saline | 0 | 7625.14 |
| Lab 6 | 34078 | Saline_M | Saline | 0 | 9628.36 |
| Lab 6 | 34084 | Saline_M | Saline | 0 | 11553 |
| Lab 6 | 34103 | Saline_M | Saline | 0 | 6138.79 |
| Lab 6 | 34105 | Saline_M | Saline | 0 | 8257.44 |
| Lab 6 | 33817 | Veh DZ_F | Vehicle Diazepam | 0 | 3083.53 |
| Lab 6 | 33873 | Veh DZ_F | Vehicle Diazepam | 0 | 5187.58 |
| Lab 6 | 33875 | Veh DZ_F | Vehicle Diazepam | 0 | 403.965 |
| Lab 6 | 34075 | Veh DZ_F | Vehicle Diazepam | 0 | 2154.03 |
| Lab 6 | 34087 | Veh DZ_F | Vehicle Diazepam | 0 | 2882.08 |
| Lab 6 | 34100 | Veh DZ_F | Vehicle Diazepam | 0 | 8970.84 |
| Lab 6 | 33830 | Veh DZ_M | Vehicle Diazepam | 0 | 2183.21 |
| Lab 6 | 33870 | Veh DZ_M | Vehicle Diazepam | 0 | 4900.47 |
| Lab 6 | 34066 | Veh DZ_M | Vehicle Diazepam | 0 | 720.38 |
| Lab 6 | 34071 | Veh DZ_M | Vehicle Diazepam | 0 | 1243.77 |
| Lab 6 | 34077 | Veh DZ_M | Vehicle Diazepam | 0 | 5835.28 |
| Lab 6 | 34083 | Veh DZ_M | Vehicle Diazepam | 0 | 723.796 |
| Lab 7 | f10-2 | Diazepam_F | Diazepam | 3 | 6232 |
| Lab 7 | f13-3 | Diazepam_F | Diazepam | 3 | 6700 |
| Lab 7 | f14-1 | Diazepam_F | Diazepam | 3 | 3990 |
| Lab 7 | f2-2 | Diazepam_F | Diazepam | 3 | 3340 |
| Lab 7 | f5-3 | Diazepam_F | Diazepam | 3 | 5219 |
| Lab 7 | f8-2 | Diazepam_F | Diazepam | 3 | 4924 |
| Lab 7 | m1-1 | Diazepam_M | Diazepam | 3 | 5609 |
| Lab 7 | m10-3 | Diazepam_M | Diazepam | 3 | 3068 |
| Lab 7 | m13-2 | Diazepam_M | Diazepam | 3 | 4614 |
| Lab 7 | m15-2 | Diazepam_M | Diazepam | 3 | 6810 |
| Lab 7 | m4-2 | Diazepam_M | Diazepam | 3 | 4117 |
| Lab 7 | m8-2 | Diazepam_M | Diazepam | 3 | 4628 |
| Lab 7 | f1-1 | MK 0.2_F | MK-801 | 0.2 | 13004 |
| Lab 7 | f11-1 | MK 0.2_F | MK-801 | 0.2 | 7331 |
| Lab 7 | f12-1 | MK 0.2_F | MK-801 | 0.2 | 13656 |
| Lab 7 | f13-1 | MK 0.2_F | MK-801 | 0.2 | 7693 |
| Lab 7 | f14-2 | MK 0.2_F | MK-801 | 0.2 | 11749 |
| Lab 7 | f15-3 | MK 0.2_F | MK-801 | 0.2 | 8542 |
| Lab 7 | f2-3 | MK 0.2_F | MK-801 | 0.2 | 6315 |
| Lab 7 | f3-2 | MK 0.2_F | MK-801 | 0.2 | 11317 |
| Lab 7 | f4-1 | MK 0.2_F | MK-801 | 0.2 | 10971 |
| Lab 7 | f5-2 | MK 0.2_F | MK-801 | 0.2 | 10305 |
| Lab 7 | f6-3 | MK 0.2_F | MK-801 | 0.2 | 6579 |
| Lab 7 | f7-2 | MK 0.2_F | MK-801 | 0.2 | 6857 |
| Lab 7 | f8-3 | MK 0.2_F | MK-801 | 0.2 | 14538 |
| Lab 7 | f9-1 | MK 0.2_F | MK-801 | 0.2 | 6685 |
| Lab 7 | m1-2 | MK 0.2_M | MK-801 | 0.2 | 8441 |
| Lab 7 | m10-2 | MK 0.2_M | MK-801 | 0.2 | 10071 |
| Lab 7 | m11-2 | MK 0.2_M | MK-801 | 0.2 | 7909 |
| Lab 7 | m12-1 | MK 0.2_M | MK-801 | 0.2 | 9965 |
| Lab 7 | m14-2 | MK 0.2_M | MK-801 | 0.2 | 7874 |
| Lab 7 | m15-1 | MK 0.2_M | MK-801 | 0.2 | 14209 |
| Lab 7 | m2-1 | MK 0.2_M | MK-801 | 0.2 | 11911 |
| Lab 7 | m3-1 | MK 0.2_M | MK-801 | 0.2 | 11761 |
| Lab 7 | m4-1 | MK 0.2_M | MK-801 | 0.2 | 10698 |
| Lab 7 | m5-1 | MK 0.2_M | MK-801 | 0.2 | 10924 |
| Lab 7 | m6-3 | MK 0.2_M | MK-801 | 0.2 | 10770 |
| Lab 7 | m7-1 | MK 0.2_M | MK-801 | 0.2 | 8072 |
| Lab 7 | m8-3 | MK 0.2_M | MK-801 | 0.2 | 12293 |
| Lab 7 | m9-3 | MK 0.2_M | MK-801 | 0.2 | 10623 |
| Lab 7 | f10-3 | MK 0.3_F | MK-801 | 0.3 | 12325 |
| Lab 7 | f11-3 | MK 0.3_F | MK-801 | 0.3 | 4907 |
| Lab 7 | f3-3 | MK 0.3_F | MK-801 | 0.3 | 9533 |
| Lab 7 | f6-1 | MK 0.3_F | MK-801 | 0.3 | 7572 |
| Lab 7 | f7-1 | MK 0.3_F | MK-801 | 0.3 | 7793 |
| Lab 7 | f9-2 | MK 0.3_F | MK-801 | 0.3 | 6545 |
| Lab 7 | m11-3 | MK 0.3_M | MK-801 | 0.3 | 7211 |
| Lab 7 | m13-3 | MK 0.3_M | MK-801 | 0.3 | 7861 |
| Lab 7 | m5-2 | MK 0.3_M | MK-801 | 0.3 | 6170 |
| Lab 7 | m6-2 | MK 0.3_M | MK-801 | 0.3 | 11398 |
| Lab 7 | m7-2 | MK 0.3_M | MK-801 | 0.3 | 7065 |
| Lab 7 | m9-1 | MK 0.3_M | MK-801 | 0.3 | 10781 |
| Lab 7 | f1-2 | Saline_F | Saline | 0 | 10028 |
| Lab 7 | f11-2 | Saline_F | Saline | 0 | 4836 |
| Lab 7 | f12-3 | Saline_F | Saline | 0 | 8319 |
| Lab 7 | f14-3 | Saline_F | Saline | 0 | 5425 |
| Lab 7 | f15-1 | Saline_F | Saline | 0 | 3990 |
| Lab 7 | f2-1 | Saline_F | Saline | 0 | 6899 |
| Lab 7 | f3-1 | Saline_F | Saline | 0 | 8023 |
| Lab 7 | f4-2 | Saline_F | Saline | 0 | 6122 |
| Lab 7 | f5-1 | Saline_F | Saline | 0 | 8517 |
| Lab 7 | f6-2 | Saline_F | Saline | 0 | 14002 |
| Lab 7 | f7-3 | Saline_F | Saline | 0 | 5559 |
| Lab 7 | f8-1 | Saline_F | Saline | 0 | 8593 |
| Lab 7 | f9-3 | Saline_F | Saline | 0 | 6318 |
| Lab 7 | m10-1 | Saline_M | Saline | 0 | 7849 |
| Lab 7 | m11-1 | Saline_M | Saline | 0 | 7361 |
| Lab 7 | m12-3 | Saline_M | Saline | 0 | 7268 |
| Lab 7 | m14-1 | Saline_M | Saline | 0 | 6232 |
| Lab 7 | m15-3 | Saline_M | Saline | 0 | 7614 |
| Lab 7 | m2-3 | Saline_M | Saline | 0 | 7158 |
| Lab 7 | m3-2 | Saline_M | Saline | 0 | 7638 |
| Lab 7 | m4-3 | Saline_M | Saline | 0 | 8349 |
| Lab 7 | m5-3 | Saline_M | Saline | 0 | 6518 |
| Lab 7 | m6-1 | Saline_M | Saline | 0 | 6401 |
| Lab 7 | m7-3 | Saline_M | Saline | 0 | 6333 |
| Lab 7 | m8-1 | Saline_M | Saline | 0 | 7048 |
| Lab 7 | m9-2 | Saline_M | Saline | 0 | 6851 |
| Lab 7 | f1-3 | Veh DZ_F | Vehicle Diazepam | 0 | 5724 |
| Lab 7 | f10-1 | Veh DZ_F | Vehicle Diazepam | 0 | 8244 |
| Lab 7 | f12-2 | Veh DZ_F | Vehicle Diazepam | 0 | 8407 |
| Lab 7 | f13-2 | Veh DZ_F | Vehicle Diazepam | 0 | 6765 |
| Lab 7 | f15-2 | Veh DZ_F | Vehicle Diazepam | 0 | 8251 |
| Lab 7 | f4-3 | Veh DZ_F | Vehicle Diazepam | 0 | 4623 |
| Lab 7 | m1-3 | Veh DZ_M | Vehicle Diazepam | 0 | 6516 |
| Lab 7 | m12-2 | Veh DZ_M | Vehicle Diazepam | 0 | 7455 |
| Lab 7 | m13-1 | Veh DZ_M | Vehicle Diazepam | 0 | 6484 |
| Lab 7 | m14-3 | Veh DZ_M | Vehicle Diazepam | 0 | 7502 |
| Lab 7 | m2-2 | Veh DZ_M | Vehicle Diazepam | 0 | 6720 |
| Lab 7 | m3-3 | Veh DZ_M | Vehicle Diazepam | 0 | 7348 |
