# Supplementary material for: Systematic assessment of the replicability and generalizability of preclinical findings: Impact of protocol harmonization across laboratory sites
Source: PLoS Biol. 2022 Nov 23;20(11):e3001886. doi: 10.1371/journal.pbio.3001886 (PMC9728859; doi:10.1371/journal.pbio.3001886)
Supplement: S2 Supplementary Stage — (DOCX) [file pbio.3001886.s002.docx]

**Table A**. Raw data and statistical results of the MK-801 treatment with the local protocol in stage 2. The p-value represent a difference from 0 for a single treatment or a difference between two treatments according to the ‘DrugTreatment’ column.

| Laboratory | DrugTreatment | mean | SE | lower.CL | upper.CL | p-value |
| --- | --- | --- | --- | --- | --- | --- |
| Lab 4 | Saline | 7.94 | 0.056 | 7.83 | 8.06 | <0.0001 |
| Lab 4 | MK-801-0.2 mg/kg | 9.11 | 0.056 | 9.00 | 9.23 | <0.0001 |
| Lab 4 | Saline - (MK-801-0.2 mg/kg) | -1.17 | 0.079 | -1.33 | -1.01 | <0.0001 |
| Lab 6 | Saline | 9.20 | 0.048 | 9.10 | 9.30 | <0.0001 |
| Lab 6 | MK-801-0.2 mg/kg | 9.52 | 0.048 | 9.42 | 9.62 | <0.0001 |
| Lab 6 | Saline - (MK-801-0.2 mg/kg) | -0.32 | 0.068 | -0.46 | -0.18 | 0.0001 |
| Lab 2 | Saline | 8.98 | 0.058 | 8.86 | 9.10 | <0.0001 |
| Lab 2 | MK-801-0.2 mg/kg | 9.46 | 0.058 | 9.34 | 9.58 | <0.0001 |
| Lab 2 | Saline - (MK-801-0.2 mg/kg) | -0.48 | 0.082 | -0.65 | -0.30 | <0.0001 |
| Lab 5 | Saline | 8.41 | 0.068 | 8.27 | 8.55 | <0.0001 |
| Lab 5 | MK-801-0.2 mg/kg | 9.21 | 0.068 | 9.07 | 9.35 | <0.0001 |
| Lab 5 | Saline - (MK-801-0.2 mg/kg) | -0.80 | 0.096 | -1.00 | -0.60 | <0.0001 |
| Lab 7 | Saline | 8.72 | 0.065 | 8.58 | 8.85 | <0.0001 |
| Lab 7 | MK-801-0.2 mg/kg | 9.09 | 0.065 | 8.95 | 9.22 | <0.0001 |
| Lab 7 | Saline - (MK-801-0.2 mg/kg) | -0.37 | 0.092 | -0.56 | -0.18 | 0.0006 |
| Lab 1 | Saline | 8.74 | 0.046 | 8.65 | 8.84 | <0.0001 |
| Lab 1 | MK-801-0.2 mg/kg | 9.55 | 0.046 | 9.46 | 9.65 | <0.0001 |
| Lab 1 | Saline - (MK-801-0.2 mg/kg) | -0.81 | 0.066 | -0.95 | -0.68 | <0.0001 |
| Lab 3 | Saline | 8.77 | 0.089 | 8.59 | 8.96 | <0.0001 |
| Lab 3 | MK-801-0.2 mg/kg | 9.27 | 0.089 | 9.08 | 9.45 | <0.0001 |
| Lab 3 | Saline - (MK-801-0.2 mg/kg) | -0.50 | 0.126 | -0.76 | -0.24 | 0.0007 |

**Table B**. Raw data and statistical results of the MK-801 treatment with the standardized protocol in stage 2. The p-value represent a difference from 0 for a single treatment or a difference between two treatments according to the ‘DrugTreatment’ column.

| Laboratory | DrugTreatment | mean | SE | lower.CL | upper.CL | p-value |
| --- | --- | --- | --- | --- | --- | --- |
| Lab 4 | Saline | 7.99 | 0.064 | 7.86 | 8.13 | <0.0001 |
| Lab 4 | MK-801-0.2 mg/kg | 8.72 | 0.064 | 8.58 | 8.85 | <0.0001 |
| Lab 4 | Saline - (MK-801-0.2 mg/kg) | -0.72 | 0.091 | -0.91 | -0.53 | <0.0001 |
| Lab 6 | Saline | 8.15 | 0.067 | 8.01 | 8.29 | <0.0001 |
| Lab 6 | MK-801-0.2 mg/kg | 9.15 | 0.067 | 9.01 | 9.29 | <0.0001 |
| Lab 6 | Saline - (MK-801-0.2 mg/kg) | -1.00 | 0.094 | -1.20 | -0.81 | <0.0001 |
| Lab 2 | Saline | 8.12 | 0.080 | 7.96 | 8.29 | <0.0001 |
| Lab 2 | MK-801-0.2 mg/kg | 8.96 | 0.080 | 8.80 | 9.13 | <0.0001 |
| Lab 2 | Saline - (MK-801-0.2 mg/kg) | -0.84 | 0.113 | -1.08 | -0.61 | <0.0001 |
| Lab 5 | Saline | 8.12 | 0.064 | 7.99 | 8.25 | <0.0001 |
| Lab 5 | MK-801-0.2 mg/kg | 9.05 | 0.064 | 8.92 | 9.18 | <0.0001 |
| Lab 5 | Saline - (MK-801-0.2 mg/kg) | -0.93 | 0.090 | -1.11 | -0.74 | <0.0001 |
| Lab 7 | Saline | 8.14 | 0.115 | 7.90 | 8.38 | <0.0001 |
| Lab 7 | MK-801-0.2 mg/kg | 8.75 | 0.115 | 8.51 | 8.99 | <0.0001 |
| Lab 7 | Saline - (MK-801-0.2 mg/kg) | -0.61 | 0.163 | -0.95 | -0.27 | 0.0011 |
| Lab 1 | Saline | 8.26 | 0.046 | 8.17 | 8.36 | <0.0001 |
| Lab 1 | MK-801-0.2 mg/kg | 9.20 | 0.046 | 9.10 | 9.29 | <0.0001 |
| Lab 1 | Saline - (MK-801-0.2 mg/kg) | -0.93 | 0.066 | -1.07 | -0.80 | <0.0001 |
| Lab 3 | Saline | 8.44 | 0.051 | 8.33 | 8.54 | <0.0001 |
| Lab 3 | MK-801-0.2 mg/kg | 9.08 | 0.051 | 8.98 | 9.19 | <0.0001 |
| Lab 3 | Saline - (MK-801-0.2 mg/kg) | -0.65 | 0.072 | -0.80 | -0.50 | <0.0001 |

**Table C**. Raw data and statistical results of the MK-801 treatment with the heterogenized protocol in stage 2. The p-value represent a difference from 0 for a single treatment or a difference between two treatments according to the ‘DrugTreatment’ column.

| Laboratory | DrugTreatment | mean | SE | lower.CL | upper.CL | p-value |
| --- | --- | --- | --- | --- | --- | --- |
| Lab 4 | Saline | 7.93 | 0.061 | 7.80 | 8.05 | <0.0001 |
| Lab 4 | MK-801-0.2 mg/kg | 8.76 | 0.061 | 8.64 | 8.89 | <0.0001 |
| Lab 4 | Saline - (MK-801-0.2 mg/kg) | -0.83 | 0.086 | -1.01 | -0.66 | <0.0001 |
| Lab 6 | Saline | 8.31 | 0.052 | 8.21 | 8.42 | <0.0001 |
| Lab 6 | MK-801-0.2 mg/kg | 9.20 | 0.052 | 9.09 | 9.31 | <0.0001 |
| Lab 6 | Saline - (MK-801-0.2 mg/kg) | -0.89 | 0.073 | -1.04 | -0.73 | <0.0001 |
| Lab 2 | Saline | 8.15 | 0.075 | 7.99 | 8.30 | <0.0001 |
| Lab 2 | MK-801-0.2 mg/kg | 8.68 | 0.075 | 8.52 | 8.83 | <0.0001 |
| Lab 2 | Saline - (MK-801-0.2 mg/kg) | -0.53 | 0.106 | -0.75 | -0.31 | 0.0001 |
| Lab 5 | Saline | 8.01 | 0.074 | 7.85 | 8.16 | <0.0001 |
| Lab 5 | MK-801-0.2 mg/kg | 9.00 | 0.074 | 8.85 | 9.16 | <0.0001 |
| Lab 5 | Saline - (MK-801-0.2 mg/kg) | -1.00 | 0.105 | -1.22 | -0.78 | <0.0001 |
| Lab 7 | Saline | 7.94 | 0.050 | 7.84 | 8.05 | <0.0001 |
| Lab 7 | MK-801-0.2 mg/kg | 8.89 | 0.050 | 8.79 | 9.00 | <0.0001 |
| Lab 7 | Saline - (MK-801-0.2 mg/kg) | -0.95 | 0.070 | -1.09 | -0.80 | <0.0001 |
| Lab 1 | Saline | 8.19 | 0.038 | 8.11 | 8.27 | <0.0001 |
| Lab 1 | MK-801-0.2 mg/kg | 9.20 | 0.047 | 9.10 | 9.30 | <0.0001 |
| Lab 1 | Saline - (MK-801-0.2 mg/kg) | -1.01 | 0.060 | -1.14 | -0.89 | <0.0001 |
| Lab 3 | Saline | 8.39 | 0.050 | 8.28 | 8.49 | <0.0001 |
| Lab 3 | MK-801-0.2 mg/kg | 9.13 | 0.050 | 9.02 | 9.23 | <0.0001 |
| Lab 3 | Saline - (MK-801-0.2 mg/kg) | -0.74 | 0.071 | -0.89 | -0.60 | <0.0001 |

**Table D**. Variance components of the Localization protocol from stage 1 (Localization 1) and stage 2 (Localization 2).

| Parameter | Localization 1 | Localization 2 |
| --- | --- | --- |
| DrugTreatment:Lab | 0.044 (28.86%) | 0.042 (29.31%) |
| Lab | 0.052 (34.68%) | 0.054 (37.67%) |
| Residual | 0.055 (36.47%) | 0.048 (33.02%) |
| Total | 0.151 (100.00%) | 0.144 (100.00%) |

**Table E.** Across laboratory analysis of the Light intensity factor as a fixed effect.

| Parameter | F-value | P-value |
| --- | --- | --- |
| DrugTreatment | 176.406 | 0.000 |
| Light Intensity | 2.663 | 0.105 |
| DrugTreatment:Light Intensity | 0.002 | 0.968 |

**Table F.** Estimated mean and 95% CI by Light intensity group.

| Light Intensity | Drug treatment | mean | lower.CL | upper.CL |
| --- | --- | --- | --- | --- |
| Bright | Saline | 8.156 | 7.985 | 8.326 |
| Bright | MK-801-0.2mg/kg | 9.005 | 8.835 | 9.176 |
| Dim | Saline | 8.104 | 7.933 | 8.274 |
| Dim | MK-801-0.2mg/kg | 8.956 | 8.785 | 9.127 |

**Table G.**  Across laboratory analysis of the Light intensity factor as a random effect.

| Parameter | Std.Dev. | Var | % |
| --- | --- | --- | --- |
| DrugDose:Lab | 0.105 | 0.011 | 14.36 |
| Lab:light_intensity | 0.012 | 0.000 | 0.20 |
| Lab | 0.153 | 0.024 | 30.67 |
| light_intensity | 0.028 | 0.001 | 1.02 |
| Residual | 0.203 | 0.041 | 53.74 |
| Total | 0.502 | 0.077 | 100.00 |

**Table H.** Across laboratory analysis of the Time of testing factor as a fixed effect.

| Parameter | F-value | P-value |
| --- | --- | --- |
| DrugTreatment | 176.405 | 0.000 |
| Time of testing | 1.499 | 0.223 |
| DrugTreatment:Time of testing | 1.076 | 0.301 |

**Table I.** Estimated mean and 95% CI by Time of testing group.

| Time of testing | DrugTreatment | mean | lower.CL | upper.CL |
| --- | --- | --- | --- | --- |
| Early | Saline | 8.127 | 7.956 | 8.297 |
| Early | MK-801-0.2mg/kg | 8.946 | 8.775 | 9.117 |
| Late | Saline | 8.133 | 7.962 | 8.303 |
| Late | MK-801-0.2mg/kg | 9.016 | 8.845 | 9.187 |

**Table J.**  Across laboratory analysis of the Time of testing factor as a random effect.

| Parameter | Std.Dev. | Var | % |
| --- | --- | --- | --- |
| DrugTreatment:Lab | 0.105 | 0.011 | 14.56 |
| Lab:Time of test | 0.056 | 0.003 | 4.08 |
| Lab | 0.148 | 0.022 | 28.88 |
| Time of test | 0.000 | 0.000 | 0.00 |
| Residual | 0.200 | 0.040 | 52.48 |
| Total | 0.510 | 0.076 | 100.00 |
