# Supplementary material for: Systematic assessment of the replicability and generalizability of preclinical findings: Impact of protocol harmonization across laboratory sites
Source: PLoS Biol. 2022 Nov 23;20(11):e3001886. doi: 10.1371/journal.pbio.3001886 (PMC9728859; doi:10.1371/journal.pbio.3001886)
Supplement: S3 Supplementary Stage — (DOCX) [file pbio.3001886.s003.docx]

**Table A**. Raw data and statistical results of the Clozapine treatments from Standardized protocol in stage 3. The p-value represent a difference from 0 for a single treatment or a difference between two treatments according to the ‘DrugTreatment’ column.

| Laboratory | DrugTreatment | mean | SE | lower.CL | upper.CL | p-value |
| --- | --- | --- | --- | --- | --- | --- |
| Lab 6 | Ultrapure water | 8.20 | 0.061 | 8.08 | 8.32 | <0.0001 |
| Lab 6 | Clozapine-1 mg/kg | 7.96 | 0.093 | 7.77 | 8.15 | <0.0001 |
| Lab 6 | Clozapine-2.5 mg/kg | 5.90 | 0.495 | 4.90 | 6.90 | <0.0001 |
| Lab 6 | Ultrapure water - (Clozapine-1 mg/kg) | 0.24 | 0.111 | 0.02 | 0.47 | 0.0364 |
| Lab 6 | Ultrapure water - (Clozapine-2.5 mg/kg) | 2.30 | 0.499 | 1.30 | 3.31 | <0.0001 |
| Lab 6 | (Clozapine-1mg/kg) - (Clozapine-2.5 mg/kg) | 2.06 | 0.504 | 1.05 | 3.08 | 0.0002 |
| Lab 7 | Ultrapure water | 8.06 | 0.047 | 7.97 | 8.16 | <0.0001 |
| Lab 7 | Clozapine-1 mg/kg | 7.35 | 0.292 | 6.76 | 7.94 | <0.0001 |
| Lab 7 | Clozapine-2.5 mg/kg | 6.06 | 0.525 | 5.00 | 7.12 | <0.0001 |
| Lab 7 | Ultrapure water - (Clozapine-1 mg/kg) | 0.71 | 0.296 | 0.11 | 1.31 | 0.0211 |
| Lab 7 | Ultrapure water - (Clozapine-2.5 mg/kg) | 2.00 | 0.527 | 0.93 | 3.06 | 0.0005 |
| Lab 7 | (Clozapine-1mg/kg) - (Clozapine-2.5 mg/kg) | 1.29 | 0.601 | 0.08 | 2.50 | 0.0379 |
| Lab 1 | Ultrapure water | 8.24 | 0.056 | 8.13 | 8.36 | <0.0001 |
| Lab 1 | Clozapine-1 mg/kg | 8.11 | 0.055 | 8.00 | 8.22 | <0.0001 |
| Lab 1 | Clozapine-2.5 mg/kg | 6.63 | 0.322 | 5.97 | 7.28 | <0.0001 |
| Lab 1 | Ultrapure water - (Clozapine-1 mg/kg) | 0.13 | 0.079 | -0.03 | 0.29 | 0.0984 |
| Lab 1 | Ultrapure water - (Clozapine-2.5 mg/kg) | 1.62 | 0.326 | 0.96 | 2.28 | <0.0001 |
| Lab 1 | (Clozapine-1 mg/kg) - (Clozapine-2.5 mg/kg) | 1.48 | 0.326 | 0.82 | 2.14 | 0.0001 |
| Lab 3 | Ultrapure water | 8.35 | 0.052 | 8.24 | 8.45 | <0.0001 |
| Lab 3 | Clozapine-1 mg/kg | 7.51 | 0.249 | 7.01 | 8.01 | <0.0001 |
| Lab 3 | Clozapine-2.5 mg/kg | 5.08 | 0.293 | 4.49 | 5.67 | <0.0001 |
| Lab 3 | Ultrapure water - (Clozapine-1 mg/kg) | 0.84 | 0.254 | 0.32 | 1.35 | 0.0020 |
| Lab 3 | Ultrapure water - (Clozapine-2.5 mg/kg) | 3.27 | 0.297 | 2.67 | 3.87 | <0.0001 |
| Lab 3 | (Clozapine-1 mg/kg) - (Clozapine-2.5 mg/kg) | 2.43 | 0.384 | 1.66 | 3.21 | <0.0001 |
| Lab 4 | Ultrapure water | 8.08 | 0.075 | 7.93 | 8.24 | <0.0001 |
| Lab 4 | Clozapine-1 mg/kg | 7.96 | 0.077 | 7.80 | 8.11 | <0.0001 |
| Lab 4 | Clozapine-2.5 mg/kg | 4.26 | 0.737 | 2.76 | 5.76 | <0.0001 |
| Lab 4 | Ultrapure water - (Clozapine-1 mg/kg) | 0.13 | 0.108 | -0.09 | 0.35 | 0.2389 |
| Lab 4 | Ultrapure water - (Clozapine-2.5 mg/kg) | 3.82 | 0.741 | 2.32 | 5.33 | <0.0001 |
| Lab 4 | (Clozapine-1 mg/kg) - (Clozapine-2.5 mg/kg) | 3.69 | 0.741 | 2.19 | 5.20 | <0.0001 |
| Lab 5 | Ultrapure water | 7.90 | 0.048 | 7.80 | 8.00 | <0.0001 |
| Lab 5 | Clozapine-1 mg/kg | 7.67 | 0.086 | 7.49 | 7.84 | <0.0001 |
| Lab 5 | Clozapine-2.5 mg/kg | 5.32 | 0.471 | 4.37 | 6.28 | <0.0001 |
| Lab 5 | Ultrapure water - (Clozapine-1 mg/kg) | 0.23 | 0.099 | 0.03 | 0.43 | 0.0224 |
| Lab 5 | Ultrapure water - (Clozapine-2.5 mg/kg) | 2.58 | 0.474 | 1.62 | 3.53 | <0.0001 |
| Lab 5 | (Clozapine-1 mg/kg) - (Clozapine-2.5 mg/kg) | 2.34 | 0.479 | 1.38 | 3.31 | <0.0001 |

**Table B.** Estimated means and 95% CI by sex per drug treatment.

| Sex | Drug treatment | mean | lower.CL | upper.CL |
| --- | --- | --- | --- | --- |
| F | Ultrapure water | 8.182 | 8.021 | 8.343 |
| F | Clozapine-1mg/kg | 7.746 | 7.495 | 7.996 |
| F | Clozapine-2.5mg/kg | 5.638 | 5.046 | 6.230 |
| M | Ultrapure water | 8.093 | 7.931 | 8.256 |
| M | Clozapine-1mg/kg | 7.764 | 7.515 | 8.013 |
| M | Clozapine-2.5mg/kg | 5.374 | 4.774 | 5.973 |

**Table C.** Across laboratory analysis of sex as a fixed factor.

| Parameter | F-value | P-value |
| --- | --- | --- |
| DrugTreatment | 64.92 | <0.001 |
| Sex | 0.58 | 0.4497 |
| DrugTreatment:Sex | 0.35 | 0.7062 |
