# Supplementary material for: Systematic assessment of the replicability and generalizability of preclinical findings: Impact of protocol harmonization across laboratory sites
Source: PLoS Biol. 2022 Nov 23;20(11):e3001886. doi: 10.1371/journal.pbio.3001886 (PMC9728859; doi:10.1371/journal.pbio.3001886)
Supplement: S1 Methods — (DOCX) [file pbio.3001886.s004.docx]

|  | **LMU** | **UBern** | **Sylics** | **TEVA** | **Orion** | **PGI** | **RUG** |
| --- | --- | --- | --- | --- | --- | --- | --- |
| **Description of video recording settings** | Trials were video-recorded with the camera placed approx. 2.5 m above the open field arena. Tests were conducted in a soundproof room.  Video-based analysis was carried out using the tracking software EthoVision XT 8.5. | Trials were recorded with IR sensitive cameras placed ca 1.5 m above the OF arena. Floor of OF arenas were backlit with IR lights. The testing was recorded and the recorders were placed outside the test room. Trials were tracked from video. | Mice were introduced into a corner of a white square open field and exploration was tracked for 10 min. | One camera above four testing arenas was used for recordings. Locomotor activity was  recorded simultaneously for four mice. | One IR sensitive camera above four testing arenas was used for recordings. Locomotor activity was  recorded simultaneously for four mice. Time for recording was 60 min. | Animal movement tracking is done through the use of three 16-beam IR arrays located in the x, y and z axis.  Tracking begins once the animal is placed in the arena and ends 15 minutes later | IR sensitive camera was place above the arena (1 arena for Stage 1 or 3 arenas for stage 2 & 3). The video acquisition was started before the mouse was places in the center of the arena and automatically stopped after 15 mins after the researcher left the experimental room. |
| **Description of behavior analysis** | Total tracking duration (center-point-detection) lasted 15 min. Tracking was started within 3 sec after the animals were placed into the arenas. Analysis was split into 5 min intervals. Parameters assessed automatically: distance; duration in center-, middle- and wall-zone; immobility. Rearing frequency was assessed manually. | Distance travelled and time spent in center during 15 minutes of OF testing were recorded. Mouse center point was detected by Ethovision and tracked automatically. Central arena was cca 22 x 22 cm. | The body reference point was tracked. To counteract the detection of distance moved due to jitter of body reference point produced by grainy video signal, track correction option in Viewer software was set to 1. | Distance moved by the animals during 15 min of the test was analysed and presented as a total distance or divided to 3-min bins | Distance moved (cm) and rotation frequency were analysed. Distance moved was analysed  in 5 min intervals as well as total distance moved in 15 min and in 60 min. Total rotation  frequency for 15 min was analysed | Distance travelled is calculated based on both the X and Y axes for positional tracking and Z axis for rearing detection. | Distance travelled was calculated taking the mouse center point as reference with the dynamic subtraction method. |
| **Description of software system** | EthoVision XT 8.5 (Noldus, RRID:SCR_000441). | Noldus Ethovision XT 11 | Viewer 2, BIOBSERVE GmbH, Bonn, Germany | Noldus, EthoVision XT15 | Noldus, EthoVision XT 14 |  | Noldus EthoVision XT 14 |
| **Bedding material** | Aspen-chip | Woodchip bedding (Lignocel JRS) | Sawdust | Aspen chip | Aspen chip | Corncob | Aspen-chip |
| **Nesting material** | 2 nestlets per cage (Ancare, Bellmore, New York, USA) | 10g sizzle pet shredded paper. |  | Paper tubes | Aspennesting material (Tapvei) | None | Shredded paper, Envirio-dry |
| **Food type** | Ssniff | Pellets, KLIBA NAFAG, Type 3430 | Harlan Teklad 2018 | Harlan Teklad 2018 | Altronim spezialfutter | Bio-serve 5001 | Pellets, Safe, Altronim |
| **Housing cage type** | Macrolon Type III | Macrolon Type III | Macrolon Type II | Techniplast Green Line GM500 | Macrolon Type III | Macrolon Type III | Macrolon Type III |
| **OF boxes (localization stage)** | White circular, 61 cm x 40.3cm | Square, 45 x 45 x 45 cm, dark grey walls, IR backlit floor (white plexiglass). | 50 x 50 cm, walls 35 cm high | Black 50 x 50 cm, walls 35 cm high | Gray, 50 x 50cm, walls 55 cm high | Square, 27.3 x 27.3cm, walls 20.3cm high | Circular, 80cm x 30cm high, dark grey walls and floor. |
| **Handling methods during experiment** | Tail handling with gloved hands | Tunnel handling during husbandry, tail handling during injection and testing. | Tail handling with gloved hands | Tail handling with gloved hands | Cupped hands during husbandry, tail handling with gloved hands | Tail handling with gloved hands | Tail handling with gloved hands |
