# Supplementary material for: Systematic assessment of the replicability and generalizability of preclinical findings: Impact of protocol harmonization across laboratory sites
Source: PLoS Biol. 2022 Nov 23;20(11):e3001886. doi: 10.1371/journal.pbio.3001886 (PMC9728859; doi:10.1371/journal.pbio.3001886)
Supplement: S1 Protocol — (DOCX) [file pbio.3001886.s005.docx]

Stage 1 Protocol per site.

**Psychogenics**

The open field (OF) chambers used were plexiglass square chambers (27.3 x 27.3 x 20.3 cm; Med Associates Incs., St Albans, VT) surrounded by infrared photobeam sources (16 x 16 x 16). The enclosure was configured to split the open field into a center and periphery zone and the photocell beams were set to measure activity in the center and in the periphery of the OF chambers. Animals having higher levels of anxiety or lower levels of activity tend to stay in the corners of the OF enclosures. On the other hand, mice that have high levels of activity and low levels of anxiety tend to spend more time in the center of the enclosure. Horizontal activity (distance traveled) and vertical activity (rearing) were measured from consecutive beam breaks. Animals were placed in the center of the OF chambers at the start of the test and remained in the chambers for 15 minutes. Total ambulatory distance, ambulatory distance in center, total rearing, and rears in the center were measured. After the 15-minute assessment was complete, the mice were returned to their home cages.

Finally for blinding, a separate RA prepared the drugs for the person running the open field.

**University of Bern**

- 45 female and 45 male C57/BL6J mice will be purchased from Janvier Laboratories, France at weaning (PND21).
- 80 animals (40 of each sex) will be used in experiment, remaining 10 animals will be used as cage-mates or extras in case any experimental animals die.
- Mice will be housed in the upstairs facility (122b) in groups of three, in Type 3 cages, with nesting material (shredded paper) and a paper tunnel. Males and females will be housed in separate housing rooms (males in room 6 and females in room 8).
- Upon arrival, mice will be allocated to cages randomly. Cages will be positioned on a Type 3 rack in 4 rows, 15 cages per sex.
- Lights will be on from 07:00 – 19:00 and off (only red lights on) from 19:00 – 07:00.
- One day after arrival, mice will be weighed and marked with a permanent marker on the tail (mouse number 1 – tail base mark, 2 – middle mark, 3 – tail tip marked).
- Throughout housing, cages and water bottles will be changed weekly. Handling during cage changes, weighing or health checks will be done by cup or tunnel handling method.
- Drug administration and OF testing will be done at age between 8-10 weeks, on two consecutive days, with each sex being tested on different days.
- Treatment allocation: Each mouse from a cage will allocated to one of the treatment groups, so that all mice in a cage receive different drug:
- 14 mice receive 0.2 mg/kg MK-801
- 6 mice with 0.3 mg/kg MK-801
- 6 mice with 3 mg/kg Diazepam
- 14 mice with saline

Cages of females positioned on a rack: Cages of males positioned on a rack:


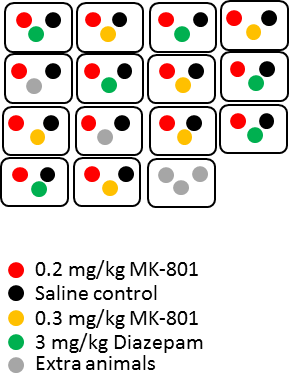

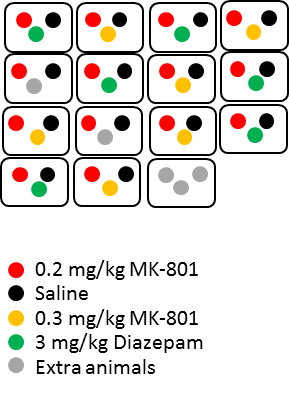


- Testing will start at 09:00.
- Drug administration will be done in the ante room, by Experimenter 1, OF testing will be done in the test room by Experimenter 2. One cage will receive i.p injections at a time, and all three animals from the cage will be tested at a single time.
- One or two days prior to testing, all mice will be re-marked for easier identification.
- Test order:
  - Cage order of drug administration and testing will be balanced by treatment:
    - There are 14 test trials per day
    - cages with six mice receiving 0.3 mg/kg MK-801 and six mice receiving Diazepam will be spread evenly across the 14 trials.
  - The order of drug administration and testing of mice within a cage will be alternated between cages, so that mice receiving the same treatment are never taken out of the cage and injected first (e.g. the order of injecting and testing in Trial 1: 0.2 MK-801, saline, diazepam; Trial 2: Saline, 0.3 MK-801, 0.2 MK-801; etc…
- Blinding:
  - All solutions will be mixed and labelled (A,B,C,D) by the third person, so that Experimenter 1 is blind to treatment. Experimenter 2 will be given a pre-determined list of trial order in which cages and mice within the cage are tested, so she will be blind to treatment allocation.

**Orion**

Animals were housed on non-reversed 12:12 light/dark cycle with ad libitum access to food and water. Open field tests were performed during the light phase, between 2 hours after lights on and 2 hours before lights off. Test setup was a large 1m x 1m grey box divided into four squared arenas 50 x 50 cm x 35 cm height. Dim lights were used in the testing room, 11 lux at the center of the arenas.

Mice were allowed to habituate to the experimental room at least for 30 min before any procedures. Thereafter, animals were given blinded treatment i.p. at a volume of 10 ml/kg. Thirty min following the injection animal was placed in the center of the open field arena and recording was started immediately. Four animals were tested simultaneously, thus one animal in each arena. The activity was recorded for 60 min by digital cameras and analysed by EthoVision XT 14 for the entire time that the animals were in the open field arena. After test was completed, the arenas were cleaned with soft tissue and water.

Males and females were tested on different days.

Blinding: One person recoded the bottles with formulations/vehicle, the second person was doing injections and the third person was doing the open field tests.

**University of Groningen**

# Material and equipment

- Open field arena (80 cm diameter x 30 cm height gray PVC)
- Top camera with IR filter connected to PC that has Media Recorder or EthoVision XT 14 (EV)
- IR lights
- Cluster music in the experimental room
- Timer
- Run sheet: date, animal information (ID, ear-clip, cage #, DOB, weight) time at the beginning and end of the test, time of dosing if any, treatment with dose and volume injected (previously BLINDED)
- Tissues and cleaning agent to clean the arenas
- Gloves & coat
- Fresh compounds to administer, syringes, needles and scale

# Procedure

Preparation:

1. Make sure the arena is clean and in the center of the camera frame. If necessary, modify the zoom and sharpness of the camera and don’t forget to use the IR filter.
2. Test that the lighting condition is good enough to track an animal all around the arena (i.e. adjust the detection settings of EthoVision until adequate detection, exposure to 2000, use dynamic subtraction model in EV).
3. Make sure the video will record at least 30 seconds before placing the animal in the arena and from then the desired duration of the test (e.g., 3’ + 15’ OFT).
4. If animals need to be habituated to the experimental room, isolate them and bring them 30 minutes before the test.
5. The waiting time after dosing has to be considered in the run sheet and must be one of the first steps in the procedure in order to save time.
6. Turn on the computer where the camera is connected, open Media Recorder and make sure all the settings are correct and type the desired name for the file (e.g OF_Tg4510_1)
7. Weight the first cage of animals and prepare the first injection (i.e., load the syringe with the corresponding compound for the first animal).
8. Once the habituation time has elapsed, inject the first animal and put it in an individual box with sawdust and lid. As soon as the animal is in the isolation cage, start the timer. (note: injections have to be done outside of the holding room to not disturb other animals)
9. While waiting for the first animal post-dosing time, prepare the injection for the second animal and if time allows (depends on the post-dosing time and the duration of the test), inject the second animal and place it in another isolation cage and start its timer.
10. Once the post-dosing time has elapsed, start the video recording and place the mouse in the center of the arena by taking it by the tail, leave the room and close the experimental room door.
11. After the testing time has elapsed stop the video if it wasn’t configured to do it automatically and take the animal to its home cage.
12. Clean the floor and walls of the arena by wetting the tissues with cleaning agent, make sure it’s dry before starting the next recording.
13. Once all animals finish, cage composition remains the same and they have to be brought back to its holding room.
14. Backup the video files (i.e., iRods, Y-drive)

When running two arenas simultaneously but in different rooms, make sure to overlap the time the animal spends in the arena in such a way that there’s a gap of at least 5 minutes between the arenas.

# Data analysis

1. Create a new project using EthoVision. Use the predefined template for circular open field with border-center zones and quadrants.
2. Adjust the arena, detection setting and analysis profile according to the desired outcome (e.g. distance traveled, time in zone)
3. Export the analysis from EV to an excel spreadsheet to perform statistical analysis and graphs.

**LMU**

**Material and equipment**

- 2 arenas
- Ethovision XT 8.5 (Noldus)
- MK801
- Diazepam
- Saline for injection
- Vehicle
- Needles
- Syringes
- Scales

**Safety requirements**

The general rules for rodent husbandry must be observed.

**Procedure**

To ensure that the assessor is blinded to treatment group the following 3 steps are performed by a technician:

- Randomize mice into treatment groups and injection order.
- Weigh mice and record.
- Calculate dose and make syringes

To be performed by investigator:

- Animals are placed in the behavior room 1hr before testing. Temperature during the experiment is same as in animal room (22±2 ᵒC).
- 2 arenas are placed on the floor and air condition is set on 1 as well as the light (20 lux). Arenas are defined in Ethovision and separated each into three zones (circles). For analysis the arena is subdivided into the zone ‘wall’ and ‘center’, comprising the outer 17% and the inner 45% of the arena, respectively.
- 30 minutes prior to OFT, the animal receives an i.p injection of either i) saline, ii) 0.2 mg/kg MK-801, iii)0.3 mg/kg MK-801, iv)3 mg/kg diazepam or v) vehicle (volume 3 ml/kg).
- Animal is placed on a 10 cm distance from the wall of each arena, faced to the wall and its movements are recorded for 15 minutes with Ethovision. Rearing is counted manually by an assessor unaware of group allocation..
- After completion of the test, the animal is placed in its home cage. The arena is cleaned with 0.1% acetic acid, after which next animal can be tested.
- Testing is performed between 09:00h and 12:00h.

**TEVA**

Animals were housed on non-reversed 12:12 light/dark cycle with ad libitum access to food and water. All the treatments were administered i.p. at a volume of 10 ml/kg.

Open field test was performed during the light phase, between 2 hours after lights on and 2 hours before lights off. Mice were allowed to habituate to the procedure room for 30 min before the treatment. Thirty min following the injection of MK801, diazepam or a vehicle, mice were placed in the open field black squared box arena. The Open field arena consisted of a large 1m x 1m box divided to 4 arenas 50 x 50 cm with 35 cm height. Four mice were tested simultaneously. Males and females were tested on different days. The activity was recorded by MediaRecorder software and digital cameras and then the videos were analyzed by EthoVision software for the entire time that the animals were in the OF arena.

**Sylics**

Mice were introduced into a corner of a white square open field (50 x 50 cm, walls 35 cm high) and exploration was tracked for 10 min (Viewer 2, BIOBSERVE GmbH, Bonn, Germany). The body reference point was tracked. To counteract the detection of distance moved due to jitter of body reference point produced by grainy video signal, track correction option in Viewer software was set to 1.
